# Update on neurobiological mechanisms of fear: illuminating the direction of mechanism exploration and treatment development of trauma and fear-related disorders

**DOI:** 10.3389/fnbeh.2023.1216524

**Published:** 2023-08-02

**Authors:** Ying Li, Weijia Zhi, Bing Qi, Lifeng Wang, Xiangjun Hu

**Affiliations:** ^1^College of Education, Hebei University, Baoding, China; ^2^Laboratory of Experimental Pathology, Beijing Institute of Radiation Medicine, Beijing, China

**Keywords:** fear extinction, medial prefrontal cortex, amygdala, hippocampus, microcircuit, BDNF, NMDA

## Abstract

Fear refers to an adaptive response in the face of danger, and the formed fear memory acts as a warning when the individual faces a dangerous situation again, which is of great significance to the survival of humans and animals. Excessive fear response caused by abnormal fear memory can lead to neuropsychiatric disorders. Fear memory has been studied for a long time, which is of a certain guiding effect on the treatment of fear-related disorders. With continuous technological innovations, the study of fear has gradually shifted from the level of brain regions to deeper neural (micro) circuits between brain regions and even within single brain regions, as well as molecular mechanisms. This article briefly outlines the basic knowledge of fear memory and reviews the neurobiological mechanisms of fear extinction and relapse, which aims to provide new insights for future basic research on fear emotions and new ideas for treating trauma and fear-related disorders.

## 1. Overview of fear

Entering the twenty-first century, mental illness has received increasing attention (Organization, 2001). Negative emotions (such as fear) are triggered not only when people experience fearful (or traumatizing) events but simply by seeing images or videos about the disasters (Pfefferbaum et al., 2014). The long-term effects of negative emotions on mental health cannot be ignored. A survey by the World Health Organization (WHO) indicates that ~70.4% of people worldwide (from 24 countries) have experienced lifelong trauma (Kessler et al., 2017). The prevalence of post-traumatic stress disorder (PTSD) in the general population is about 6%, increasing to 25% in groups that have experienced severe trauma (Ressler et al., 2022). Nowadays, the occurrence of trauma and fear-related disorders, represented by depression, anxiety disorders, and PTSD, has become a common phenomenon due to the increasing pressure of life, the gradual increase of local wars and social violence, and the frequent occurrence of accidents such as major traffic accidents and natural disasters (Organization, 2001). To some extent, the presence of fearful emotions is a protective mechanism for the organisms to adapt to the complex environment to survive (Mobbs et al., 2015). For example, they warn individuals about dangerous situations or protect them from secondary harm. However, excessive fear memory triggers several mental disorders (Rajbhandari et al., 2018). A common feature of those mental disorders is the inability to extinguish the fear memory (Plutchik et al., 1979; Mobbs et al., 2015). Therefore, research on the mechanisms of fear extinction will profoundly impact the treatment of trauma and fear-related disorders and human mental health (Hagihara et al., 2021).

The conditioned fear model is a classic animal model for studying fear memory, focusing on acquiring, storing, retrieving, extinguishing, and generalizing fear memory (Vervliet and Geens, 2014). The model is based on the classical Pavlovian conditioning. In this model, a neutral stimulus (e.g., sound, conditioned stimulus, CS) is paired with an aversive stimulus (e.g., electric shock, unconditioned stimulus, US) multiple times. Individuals learn that the CS predicts the presence of the US, forming a CS–US fear memory. Subsequently, the individual's fear response (also named conditioned response, CR) is triggered by the CS alone. If the CS appears repeatedly and is no longer reinforced by the US, the extent to which the CS triggers a CR will gradually diminish or even disappear, a process known as fear extinction. Using the conditioned fear model and its variants can help gain insight into the mechanism of fear memory extinction, clarify the critical targets for regulating fear memory, and explore effective methods for eliminating the original fear memory association and creating a new secure memory association.

Biologists have summarized the five sequential biological responses that individuals may experience in response to the fearful stimulus: freezing, flight, fight, fright, and fainting (Gray, 1987; Bracha, 2004; Stefan Bracha et al., 2004; Hunt, 2007). The occurrence of this sequence is closely related to the level of fear (Bracha, 2004; Roelofs, 2017). More specifically, when presented with danger, rodents' natural reaction to fear is to “freeze,” a behavior in which an animal does not move except to breathe (Johansen et al., 2011). This behavior helps reduce the chance of predators detecting the prey and increases survival (Fanselow, 1980; McSweeney and Murphy, 2014). In most studies on conditioned fear, the duration of freezing when an animal receives a CS often indicates the degree of fear in rodents (Zelikowsky et al., 2012). Freezing in rodents corresponds to what clinicians describe as “hypervigilance” in diagnosing human fear responses. This means that freezing in rodents corresponds to stopping and observing the environment as for humans (Gray, 1987; Bracha, 2004). Subsequently, rodents activate active fight-or-flight reactions accompanied by parasympathetically dominated heart rate deceleration (Roelofs, 2017). Several studies also regard the above two actions as indicators of fear (De Franceschi et al., 2016; Guo et al., 2021; Trott et al., 2022; Campagner et al., 2023). Fright manifests as tonic immobility or “playing dead” in the early stages. In child psychology, tonic immobility is often confused with freezing, as both exhibit a stationary state. However, the former is thought to occur when there is direct contact with a predator or dangerous condition, whereas the latter is the initial manifestation of facing fear signals (Bracha, 2004). In addition, fainting is difficult to investigate and is therefore hardly studied (Bracha, 2004).

In summary, this review briefly outlines the basic knowledge of fear memory. It aims to provide new ideas and insights for subsequent research by reviewing studies on the neurobiological mechanisms of fear, including studies on neural circuits and molecular mechanisms.

## 2. Introduction of fear memory

### 2.1. Fear memory model and its variants

Although existing models are numerous and widely used, no strict classification is established between models of fear memory, and the different models are always a blend of sensitization theory and fear conditioning (FC) theory (Bienvenu et al., 2021). Sensitization means that when the stimulation of neurons is accompanied by aversive stimulation given to experimental animals, and whenever a weaker aversive stimulus is present, the neurons will also show strong intense stimulation (Kupfermann and Kandel, 1969). The inescapable shock PTSD model is typically one of the classical models based on sensitization theory (Pynoos et al., 1996). FC models can reflect the core manifestations of fear and observe the triggers of fear onset and extinction and the accompanying changes in animal behavior. Moreover, many studies have been conducted to manipulate the process of fear acquisition and extinction based on FC models. Studies are attempting to characterize the changes in brain regions, circuits, and molecular mechanisms involved in the process of fear extinction and further construct a whole-brain model of fear extinction using pharmacological interventions, gene editing, chemical genetics, and optogenetics. Several commonly used models of fear are briefly described below.

The foot-shock (FS) model uses electric shocks (both plantar and tail shocks) to induce a sense of existential threat in the animal, generating a strong sense of fear and thus exhibiting a fear response (Pynoos et al., 1996). This model is currently one of the most important animal models for studying trauma and fear-related disorders and usually combines US (e.g., electric shock) with CS (e.g., sound or light) given simultaneously and repeatedly (Servatius et al., 1995). FS models include the contextual FS model and the cued FS model. Lin et al. used the FS model to reveal single multimodal stress-activated brain circuits through c-Fos mapping. They showed that the paraventricular nucleus (PVN) and the bed nucleus of the stria terminalis (BNST) of the hypothalamus are the central stress-related brain regions (Lin et al., 2018). Researchers have created various targeted animal models by varying the parameters of the FS model (e.g., anxiety and panic model, PTSD model, and operant conflict model; Bali and Jaggi, 2015). However, real-world stressors are complex and diverse (Cassidy, 2022). It is unrealistic to explore all possible stressors. Additionally, the more complex the stressors, the more difficult it is to tease apart the neurobiological changes. Although plantar or tail electroshock may not be adequate to reveal the complete spectrum of trauma and fear-related disease features in real-life circumstances, those simple models can be adequate to lead to an increased understanding of specific aspects of fear and the underlying neurobiology mechanism (Whitaker et al., 2014).

Social stress is one of the most critical variables in the etiology of stress-related diseases (Adamec and Shallow, 1993). Poor social relationships and repeated failure experiences are closely related to prolonged stress exposure, accompanied by various stress-related behaviors, such as social avoidance, learned helplessness, and sleep disturbances (McEwen and Gianaros, 2010; Lucas-Thompson and Goldberg, 2011; Lo Martire et al., 2020). Therefore, social stress models, such as the repeated social defeat stress (RSDS) model and the predator-based psychosocial stress (PPS) model, have been used to study the neurobiological mechanisms of stress-related disorders (e.g., social anxiety disorder). Among them, the PPS model is based on the natural food chain relationship between animals, in which subjects are directly exposed to a hungry predator (e.g., a caged mouse facing a hungry cat) or indirectly exposed to the odor of a predator (e.g., a mouse touching or smelling the feces of a cat or fox), and thus feel a sense of existential threat that leads to great fear (Servatius et al., 1995; Lindström et al., 2018). The RSDS model has been used to study individual differences in stress sensitivity and general stress-related emotion disorders (Krishnan et al., 2007).

Single prolonged stress (SPS) models include the water immersion and restraint stress (wIRS) model and the chronic restraint stress (CRS) model (Liberzon et al., 1997). The classical SPS model was constructed by subjecting the animals to three stressors (physical restraint, forced swimming, and exposure to either) one by one and then entering a resting period (no stimulation) for the next 7 days to establish an FC model (Yamamoto et al., 2009; Winters et al., 2021). It is noteworthy that this minimum of 7 days of social isolation is necessary for the effects seen following SPS (Knox et al., 2012; Lisieski et al., 2018). Several researchers have chosen the SPS model as one of the most often used rodent PTSD models, because of its capacity to simulate the altered hypothalamic-pituitary-adrenal (HPA) axis in PTSD patients (Yamamoto et al., 2009; Knox et al., 2012). Among this, the wIRS was first designed by Yoneda et al. (1980) to simulate the sudden, brief traumatic experience in water and was applied to the development of stress medications. The specific experimental process is as follows: experimental animals are fasted overnight, placed in metal cages, and immersed in 25°C water for 3 h. Behaviors shaped by this model (e.g., freezing and hopelessness) are similar to those of patients suffering from trauma or stress-related disorders and persist for 2 weeks after the stress. The model mimics people's fear when their safety is threatened and is closer to the pathogenesis of psychiatric disorders such as phobias. Recently, Richter-Levin has developed an underwater trauma (UWT) model that is more consistent with the symptoms of trauma and stress disorders and has been used to study the neural mechanisms of different subregions and circuits of the HPC during fear extinction (Richter-Levin, 1998; Zuo et al., 2022).

In addition to the above models, fear-potentiated startle (FPS) and inhibitory avoidance (IA) models have also been used in fear-related studies. Researchers usually choose different models according to the purpose of the study. However, there is still room for improvement in the design and procedures of existing models: current models are widely used to explore the processes of memory and emotions and lack targeted applications. For example, the FS model is not only used in fear studies but is also the mainstream model for depression and anxiety studies. A long-term misconception of how the field uses FS to study different processes is that the initial FS stress may be similar. Still, experiments following the FS often vary depending on which emotional process is being studied. In other words, different protocols are used to study different aspects of fear extinction and emotion (i.e., open field for anxiety, but tail hang for depression, etc.). In addition, different brain regions/molecular mechanisms implicated in distinct behaviors are being studied. Second, the existing models need more detailed and standard procedures, for example, how different intensities and durations of plantar electroshock affect the construction of fear-related models. Overall, fear-related models and their variants are still being updated and iterated.

### 2.2. Processes of fear memory

The FC model divides the fear process into several parts: acquisition, consolidation, retrieval, extinction, and generalization. Specifically, with the repeated association of CS and US pairings, subjects will enter the acquisition phase of fear memory (Aubry et al., 2016). Once a stable association is established, the short-term fear memory becomes a potential long-term memory, which is the consolidation process (Schafe et al., 2001). Two processes after memory consolidation are reconsolidation and extinction (Tronson et al., 2009). For the reconsolidation process, traditional memory consolidation theory suggests that once newly formed memories are consolidated, they cannot be easily erased or changed (Schiller et al., 2010). However, memories are usually reconsolidated more than once, implying that memory consolidation is not a one-time process and can only occur after stabilizing protein synthesis (Nader et al., 2000; Kindt and Soeter, 2013). When subjects are re-exposed to the same, single CS or a similar environment, they may undergo the process of extinction and establish new memory traces (Gu et al., 2022; Hua et al., 2023). On the other hand, fear generalization refers to extending the learned fear experience to other relevant stimuli or contexts (Dunsmoor et al., 2011). From an adaptive perspective, fear generalization during memory retrieval enables better responses to dangerous situations. In contrast, non-adaptive fear generalization may induce overreaction (e.g., perceiving safe stimuli as threatening cues), leading to severe mental disorders (Dunsmoor and Paz, 2015).

### 2.3. Categories of fear memory

#### 2.3.1. Cued and contextual fear

As mentioned above, neutral stimuli such as sound and light are usually considered as CS, and aversive stimuli such as plantar electric shock as US. During training, CS and US are usually presented in pairs several times, and the animals learn not only CS–US associations but also US-context associations. Therefore, CS and context acquire predictive value and trigger CR in the animal even in the absence of US. The former is called cued conditioned fear memory and the latter is called contextual conditioned fear memory. Cued and contextual processes are the two main components of fear memory (Curzon et al., 2009). Among this, contextual conditioned fear memory is more complex because the environment responds to a combination of stimulus elements, where olfactory, visual, tactile, temporal, and spatial elements are integrated into a holistic representation (Maren et al., 2013; Chaaya et al., 2018). The hippocampus (HPC) is currently considered a key brain region for contextual fear memory (Lehmann et al., 2009; Chaaya et al., 2018; Jimenez et al., 2020). During the acquisition, retrieval, and extinction of conditioned fear memory, spatial information from the surrounding environment is integrated during learning to form long-term contextual memory (Maren, 2011). Individuals must first form a contextual representation during this process. The HPC, which encodes and retrieves contextual information and forms contextual representations, is an important part of the contextual memory neural circuit and plays an important role in storing specific memories. Damage to the HPC causes a reduction in the “freezing” response of rodents to electroshock-matched contexts (Kim and Fanselow, 1992). At the same time, the HPC can also form representations across different experiences, linking objects and events to the time, space, and environment in which they occur and forming overall environmental representations, including olfactory and visual elements, at the population cell level (Maren et al., 2013). Neural network models by Schapir et al. identified different learning systems within the HPC, dentate gyrus (DG) granule cells translate rich inputs from the internal olfactory cortex into sparse outputs that project to the CA3 pyramidal cell network, and CA3 pyramidal neurons project to the CA1 (Schapiro et al., 2017). The trisynaptic circuit from cortex to DG and from CA3 to CA1 helps maintain contextual memory-specific representations. CA1 receives both CA3 memory-extracted projections and cortical perceptual input and is also necessary for contextual inputs (Schapiro et al., 2017; Sans Dublanc et al., 2020). Thus, the monosynaptic pathway from the cortex to CA1 facilitates the formation of a holistic representation of the environment.

#### 2.3.2. Innate and learned fear

Fears can be classified as innate and learned. In contrast to learned fears, innate fears do not depend on direct injurious experiences or associative learning processes (Silva et al., 2016). Survival-related stimuli, such as predators, height, and pain, trigger innate fear responses. Learned fear is an associative learning process. Learning occurs when an aversive stimulus (e.g., pain or a novel US) is linked to a neutral stimulus (e.g., sound). Learned fear can not only be acquired based on repeated and simultaneous associations between both stimuli, but also with a single CS and US pairing, and the pairing does not need to be simultaneous (e.g., trace conditioning; Raybuck and Lattal, 2014). Innate and learned fear also differ in their neural mechanisms (Gross and Canteras, 2012). For example, the lateral habenula (LHb) to laterodorsal tegmental nucleus (LDT) pathway plays a decisive role in the innate fear induced by the odor of natural enemies (Pereira and Moita, 2016). In addition, even the subclasses of innate fear do not recruit the same neural circuit. For instance, fear of aggressive members of the same species or predators and two subcategories of innate fear share the circuits between AMY and cortex. While the former mainly relates to the connection between the olfactory bulb or accessory olfactory bulb and the posteroventral part of medial AMY, the latter depends more on the posterodorsal part (Kollack-Walker et al., 1999).

#### 2.3.3. Recent and remote fear

Fears can be classified as recent and remote fear according to the time course of fear memory. When first exposed to a threat, subjects react immediately behaviorally and enter a cognitive process of encoding and consolidation, mainly at the memory level. This process includes two forms of interactive memory consolidation: synaptic consolidation and systemic consolidation. The former involves changes at the short-term synapse level, while the latter contains changes in long-term brain regions (Shi et al., 2018). Recent fear memories that were initially kept in the HPC progressively move to the cortex and develop into remote fear memories as memory processing becomes more complex and storage duration rises (Frankland and Bontempi, 2005; Corcoran et al., 2013). As a result, recent fear memory usually develops in several hours, while remote fear memory typically takes at least 12 days to manifest (Dixsaut and Gräff, 2021). Studies have shown that the brain networks and neural circuits involved in recent fear memory have been initially identified, and the cognitive neural mechanisms of remote fear memory are gaining attention. For instance, local lidocaine injections in the anterior cingulate cortex (ACC) before memory recovery can inhibit the retrieval of remote rather than recent memories (Frankland et al., 2004). Although both recent and remote fear requires the involvement of BLA, as shown by the convergent storage pattern of BLA neurons (Liu et al., 2022), Do-Monte et al. (2015) covers a pathway for remote fear memories that is BLA independent [mPFC–paraventricular nucleus of the thalamus (PVT)–central amygdaloid nucleus (CeA); Do-Monte et al., 2015]. This result suggests that distinguishing and clarifying the neural mechanisms underlying recent and remote fears is just around the corner. Excitingly, by using techniques such as chemical genetics and optogenetics, studies have found that the extinction of remote fear memory can be successfully facilitated by modulating specific brain regions (e.g., HPC) and neural circuits [e.g., infralimbic cortex (IL) → thalamic nucleus reuniens (NRe) → BLA circuits; Ishikawa et al., 2016; Silva et al., 2021]. Specifically, during recent fear memory extinction, neurons with IL projections to the BLA were heavily activated, whereas neurons in the NRe projecting to the BLA and IL neurons projecting to the BLA did not show significant activation. During remote fear memory extinction, although the neurons projecting from IL to BLA were not significantly activated, the NRe brain region served as a “bridge.” The neurons projecting from IL to NRe and from NRe to BLA were activated, suggesting that the IL → NRe → BLA neural loop mediates the extinction of remote fear memory (Silva et al., 2021).

#### 2.3.4. Auditory, visual, and olfactory fear

Based on the sensory input channel to gather the fear information, fear memory can be divided into auditory, visual, and olfactory fear. The auditory conditioned fear (AFC) model uses various tones as CS and is widely used to establish the conditioned fear model in rodents. First, the model is relatively standardized and straightforward in its operational procedures. Researchers can directly explore discriminative learning by selecting different tones as CS (Antunes and Moita, 2010). Second, the associations established by the AFC usually last from a few hours to several months and are relatively valid and long-lasting (Pickens et al., 2009). Third, the AFC model derived from Pavlov's dog problem has a long history of value. The visually conditioned fear model, often used to evoke innate fear, induces a sense of fear by mimicking the visual stimulus of a predator sweeping overhead. In rodents, the suprachiasmatic nucleus (SC) of the thalamus-lateral posterior nucleus (LP)–lateral amygdala (LA) circuit is specialized to process information closely related to survival. It is essential to the one set of innate defensive responses (e.g., avoidance) corresponding to visual threats (Wei et al., 2015). Although using upper visual field shadows to induce fear in mice has been effective (Wei et al., 2015), mice do not have sensitive visual perception (Yang et al., 2016). It was found that mice rely primarily on smell and whiskers to discriminate their surroundings (Yang et al., 2016). For instance, placing a drop of a component derived from fox urine in mice's active area can induce freezing behavior and increased heart rate (Yang et al., 2016). In addition, rodents are also sensitive to several odors, such as urinary protein homologs from cat fur (Papes et al., 2010), thiazoline-related innate fear-eliciting compounds (tFOs; Matsuo et al., 2021), and 2-phenylethylamine in carnivore urine (Ferrero et al., 2011). Olfactory signals cause fear in a wide range of animals. Fish, for instance, may detect fear by smell. Injured zebrafish will expel an alarm chemical into the water from their skin. When others smell this material, it will set off their terror response, causing them to behave erratically or freeze (Akinrinade et al., 2023). The connectivity structure of olfactory neurons has now been clarified. Chen et al. (2022b) found that the processing of olfactory information involves the olfactory bulb, which perceives olfactory information from the nose; the piriform cortex, the main olfactory processing center; and several other regions that receive information from the olfactory bulb. Along the direction of the piriform cortex's anterior–posterior axis, the olfactory neurons' projections form a triadic loop structure. Such a structure suggests that these non-interfering, relatively parallel neural circuits are likely to form a division of labor, each processing different elements of olfactory information (Chen et al., 2022a). Evidences also highlights the important role of basolateral amygdala (BLA) in the process of olfactory fear conditioning. For example, injection of AP5 (N-methyl-D-aspartate receptors (NMDARs) antagonist) into the BLA before training disrupted fear conditioning to the odor (Walker et al., 2005).

#### 2.3.5. Social fear

Researchers use several social fear models (three-chamber social preference test, resident intruder test, social preference-avoidance test, and ultrasonic vocalizations recording) as research models, treat fear signals transmitted by partners as US and neutral stimuli as CS, and reveal the mechanism of dangerous information transmission in the group (Rammal et al., 2010; Toth and Neumann, 2013; Debiec and Olsson, 2017; Kalman and Keay, 2017; Ni et al., 2020; Qi et al., 2022; Montag et al., 2023). Among this model, the three-chamber social preference test is the classical one to test the social state (Pearson et al., 2010). The method was designed based on the characteristics of mice that have a natural preference for group living, sensitivity to the external environment, and the desire to explore new objects (Jabarin et al., 2022). The procedure is simple and easy, and can quickly quantify the social status of mice. Thus, it is widely used to assess the social behavior of normal mice and changes in social behavior in disease models. But the method converts difficult-to-quantify social behaviors such as sniffing and chasing among mice into an assessment of the activity time and distance in a specific area (Pearson et al., 2010). In addition, Zheng et al. (2021) developed a social fear conditioning based on operant conditioning. This social fear conditioning consists of four phases: housing acclimation, conditioning apparatus habituation, conditioning, and behavioral testing. During housing acclimation, mice will be converted from multi-cage feeding to single feeding to adapt to the animal feeding environment after the model is established. During conditioning apparatus habituation, mice will freely explore the modeling environment for 10 min to understand that the modeling environment is safe, which allows the mice to better establish the fear of social stimuli rather than the environment. During the conditioning phase, mice were given plantar shocks whenever they socialized with the stimuli. During behavioral testing, mice were tested for social fear manifestations using similar metrics to the three-chamber social preference test. Mice that experienced social fear conditioning showed solid and sustained social fear and social avoidance responses to other mice (Zheng et al., 2021). Compared to traditional models, the conditioned social fear model has the advantage that fearful mice do not exhibit generalized anxiety and depression-like behaviors, making it a suitable animal model for studying the neural mechanisms of social fear (Zheng et al., 2021). Previous studies have emphasized the important role of somatostatin (SST) and parvalbumin (PV) interneurons in social fear. For instance, NL3R451C knock-in (social deficit) mice have low NMDARs function in the mPFC resulting in reduced activity of PV interneurons, which leads to abnormalities in the processing of social information in the prefrontal cortex. D-cycloserine (DCS), a partial agonist of NMDARs, can restore NMDARs function and PV neuron activity, thereby restoring the social deficit in the mouse model (Cao et al., 2018). In addition, it is shown that direct and indirect social fear learning has indistinguishable behavioral effects and involves the participation of shared underlying fear learning neural networks [including AMY, anterior insula (AI), and ACC; Lindström et al., 2018]. Evidence from zebrafish suggests that different types of learning (social vs. non-social learning) involve the activation of other brain regions (Pinho et al., 2021). Social learning is related to olfactory bulbs, ventral zone of ventral telencephalic area, ventral habenula, and ventromedial thalamus, whereas asocial learning recruits dorsal habenula and anterior tubercular nucleus (Pinho et al., 2021).

## 3. Brain morphology study of fear extinction

During the extinction process of fear memory, individuals show different extinction characteristics, which may be caused by individual differences and can be explained by morphological features of the brain (Ehlers et al., 2020; Fraenz et al., 2020). Brain morphology refers to measuring brain structure, including volume and shape. Local morphological changes during development, aging, and disease are typically compared to control groups to determine how these biological changes can lead to deleterious effects. Those indicators are usually used to compare the brains of specific groups with controls to determine differences between groups (Madan, 2017).

Using techniques such as structural magnetic resonance imaging (MRI) and diffusion tensor imaging (DTI), existing studies have shown morphological changes in the brain during fear memory extinction. Fourteen subjects were recruited for a 2-day experiment on fear memory extinction. The experiment measured and analyzed the skin conduction response (SCR) index, which reflects the behavior of fear memory extinction, and the MRI index, which can display structural images and measure cortical thickness. The results showed that the thickness of the ventromedial prefrontal cortex (vmPFC), especially the medial orbitofrontal cortex (mOFC), was positively correlated with the SCR during the extinction process (Milad et al., 2005). The thicker the vmPFC, the faster the extinction learning occurs. Similar results have been found in other studies (Winkelmann et al., 2016). Even though the research mentioned above implies that changes in brain morphology may alter the process of fear memory extinction, the result has yet to reach a consensus. More research is needed before any conclusions can be formed. According to Ehlers and his colleagues, no correlation was found between the AMY volume and the mOFC thickness during fear memory extinction in a 107-healthy adult sample (Ehlers et al., 2020). They suggested that the correlation results found in previous studies came from small, simple studies (e.g., only 14 participants participated), which may imply a sampling bias (Ehlers et al., 2020).

## 4. Neurobiological mechanisms of fear extinction

### 4.1. Key brain regions and their neural circuits in fear extinction

Most research on the neurological foundations of Pavlovian fear conditioning has mainly concentrated on rodents. The brain circuits of several typical fear memories have been identified. For example, the auditory conditioned fear memory neural circuit is that auditory information is transmitted to excitatory neurons in the LA via the auditory cortex and nuclei, such as the medial geniculate nucleus of the thalamus, and then project directly to the CeA or indirectly to the CeA via the BA, and finally by inhibitory interneurons in the CeA downstream to the brainstem [e.g., parabrachial nucleus and periaqueductal gray matter (PAG)] and the thalamus [e.g., medial geniculate nucleus (MGN)], resulting in fear-related behavior (Dejean et al., 2015). The visually conditioned fear memory neural circuits consist of two mutually independent neural pathways (Robson and Hall, 1976; Tamietto and de Gelder, 2010). The high cortical path refers to the transmission of visual information from the retina through the lateral geniculate nucleus (LGN) of the thalamus to the striate cortex (also known as the primary visual cortex, V1) for fine analysis, followed by the transmission of the processed signal to the association cortex and finally to the AMY, which regulates the production of fear-related emotions. The low subcortical path is the visual pathway that allows danger signals to pass through the direct projections between the superior colliculus and LGN and finally to the AMY to produce the fear response (Johnson, 2005; Tamietto and de Gelder, 2010). With the plantar electroshock, as in the US, fear conditioning reaches the AMY via the somatosensory thalamocortical relay (e.g., ventral posterior nucleus) and the brainstem (Bouton et al., 2021).

Fear extinction requires special AMY and prefrontal cortex circuits but overlaps similar brain regions with fear acquisition and expression. Fear extinction circuits may inhibit fear expression circuits from suppressing the fear response (Dejean et al., 2015). It is generally accepted that the neural network of fear extinction consists mainly of the mPFC, the AMY, and the HPC (Park and Chung, 2019; Gu et al., 2022). The three brain regions mentioned above and their neural circuit mechanisms will be the main topic of the following section.

#### 4.1.1. Medial prefrontal cortex

The mPFC is extensively connected to cortical and sub-cortical areas (Porter, 2020) and is critical for cognition, emotion, and motivation (Rogers et al., 2004; Lapiz and Morilak, 2006). Among them, studies focusing on the role of the mPFC in fear extinction processes originated in the sensory cortex (Teich et al., 1989; Falls and Davis, 1993). LeDous et al. initially hypothesized that rats with visual cortex lesions had considerably prolonged extinction processes, indicating impaired extinction of fear memories (LeDoux et al., 1989). The ventral medial prefrontal cortex (vmPFC) has since been linked to a function in fear extinction in a steadily rising number of studies. For example, disruption of the vmPFC in rats before extinction training resulted in increased freezing behavior and impaired extinction memory 24 h later (Quirk et al., 2000).

For rodents, mPFC is composed mainly of the ACC, prelimbic cortex (PL), and IL (Porter, 2020; Dixsaut and Gräff, 2021). PL and IL play distinct roles in the FC process: PL receives inputs from fear neurons and controls innate fear expression (Knapska et al., 2012). IL mediates extinction by projecting to BLA (Bloodgood et al., 2018; Lingawi et al., 2019). For instance, mice that underwent fear extinction training and successfully extinguished their fear exhibited increased neuronal excitability in IL–BLA projection neurons (Bloodgood et al., 2018). The rodent IL is widely considered a homologous structure of the vmPFC of the human brain (Myers-Schulz and Koenigs, 2012). Inhibition of mPFC submarginal area (e.g., IL) activity interferes with the consolidation of fear extinction memory without affecting the acquisition of extinction memory; stimulation of IL neurons attenuates CeA area neuronal activity, reducing the fear response (Quirk et al., 2006). It was shown that the level of freezing behavior was significantly reduced in rats stimulated with 100 Hz electrical stimulation of the IL within 100–400 ms after the extinction recall phase in CS (e.g., providing a sound cue). In contrast, stimulation of PL showed the opposite effect (Vidal-Gonzalez et al., 2006). In addition, exposure to the SPS model may lead to decreased intrinsic membrane excitability and firing rate of the IL, resulting in PTSD-like symptoms (Nawreen et al., 2021). Canto-de-Souza et al. demonstrated that mice exhibited reversibility of impaired extinction memory after experiencing the SPS model by daily optogenetic stimulation (20 Hz, 2 s stimulation every 10 s for 15 min/day for 7 days) of excitatory neurons of the left but not the right IL (Canto-de-Souza et al., 2021).

Relevant meta-analytic studies have clarified brain regions that overlap with fear extinction in tests of extinction recall (Fullana et al., 2018). Moreover, empirical studies further support vmPFC as a critical brain region for memory extinction (Greco and Liberzon, 2016). However, when directly comparing threat stimuli (CS) that have extinguished with unextinguished (animals acquired two CS, but only one of them was presented during extinction), consistent activation is observed in the dorsolateral prefrontal cortex (dlPFC) as well as the HPC. One possibility is that dlPFC in extinction recall has a similar role to vmPFC in extinction learning, for example, suppressing subcortical responses; another possibility is that extinction memory retrieval during recall depends on dlPFC (Fullana et al., 2018). Neural circuits composed of different brain regions are involved in different stages of fear extinction. Recently, it has been proposed that extinction is facilitated by enhancing the vHPC to PL connection. For example, activation of the vHPC–PL circuit using optogenetic techniques after repeated fear extinction training facilitated the extinction of fear memory. However, this facilitation effect was only present in mice undergoing long-term extinction training. Stimulation of mice that had experienced only one extinction training session impaired the extinction of fear memory (Szadzinska et al., 2021). This result highlights the change of the vHPC–PL circuit during fear extinction training and further talks about the balance of the vHPC to PL vs. BLA to PL inputs and how they might be dynamically regulated during fear extinction. Another result supports that the connectivity of the BLA–mPFC circuit has different roles in promoting or impairing fear memory extinction and supporting brain lateralization (Vafaei et al., 2022). Mice were infused with corticosterone into the IL when the unilateral BLA inactivated impaired fear memory extinction. However, the extinction effect is absent when the BLA is inactivated bilaterally. This is because the BLA–IL pathway is completely blocked in the case of bilateral BLA inactivation and cannot exert the extinction effect (Vafaei et al., 2022). The mechanism by which the BLA–mPFC circuit is involved in fear memory extinction may involve the following processes: CS emergence, mPFC, excitation of related cortical and thalamic, activation of inhibitory interneurons in the AMY, GABA release-reduced excitability of intra-amygdala projections to CEA neurons, reduced CEA output, and inhibition of conditioned fear behavior expression. Electrophysiology evidence also supports the AMY–HPC–mPFC circuit's vital role in fear extinction. This circuit exhibits reduced theta power and synchrony after fear extinction (Hill and Martinowich, 2016). Specifically, a phase reset occurs in the prefrontal cortex after successful fear memory extinction and directs changes in the AMY theta wave, thereby synchronizing theta oscillations in the prefrontal cortex (Lesting et al., 2013; Likhtik et al., 2014). This synchronization means that the AMY receives fear-inhibitory signals from the prefrontal cortex (Lesting et al., 2013).

The brain's normal functioning requires maintaining a delicate balance between excitability and inhibition. Therefore, the cerebral cortex has evolved GABAergic interneurons that play different roles based on differences in unique molecular characteristics, morphological features, and electrophysiological properties (Schuman et al., 2019). Sun et al. used monosynaptic rabies virus tracers in combination with fluorescence micro-optical tomography to map the whole brain of remote inputs of GABAergic interneurons in the mPFC (Sun et al., 2019a). Among them, GABAergic neuronal subtypes in mPFC mainly included PV^+^ neurons and SST^+^ neurons, expressing vasoactive intestinal peptide (VIP^+^; Ährlund-Richter et al., 2019; Sun et al., 2020). During fear extinction, PV^+^ interneurons in the mPFC form inhibitory synapses with the principal neurons and silence them, thereby promoting fear extinction (Tsvetkov et al., 2002). The plasticity of the PV network in IL mPFC is involved in the top-down control process of fear extinction. SST^+^ targets the apical dendrites of pyramidal neurons and is essential in regulating dendritic activity and synaptic plasticity. It was shown that activation of SST^+^ interneurons 30 min before fear extinction increased freezing behavior in mice using chemical genetics, and inactivation of SST^+^ interneurons decreased freezing behavior (Xu et al., 2023). This may be because the activation of SST^+^ interneurons affects the elimination of dendritic spines on the same dendritic branch in the face of different FCs. In addition, SST^+^ interneurons have adolescent-specific plasticity, as evidenced by increased SST^+^ interneuron-pyramidal GABAergic transmission and specifically enhanced synaptic inhibition during puberty. This enhanced inhibition cannot be restored by extinction training. VIP^+^ neurons can be activated by vHPC input and inhibit cortical pyramidal neuron activity (Lee et al., 2019). VIP^+^ interneurons can effectively control the circuit representation of mPFC–HPC and drive avoidance behavior (Lee et al., 2019).

#### 4.1.2. Amygdala

**Figure 1:**
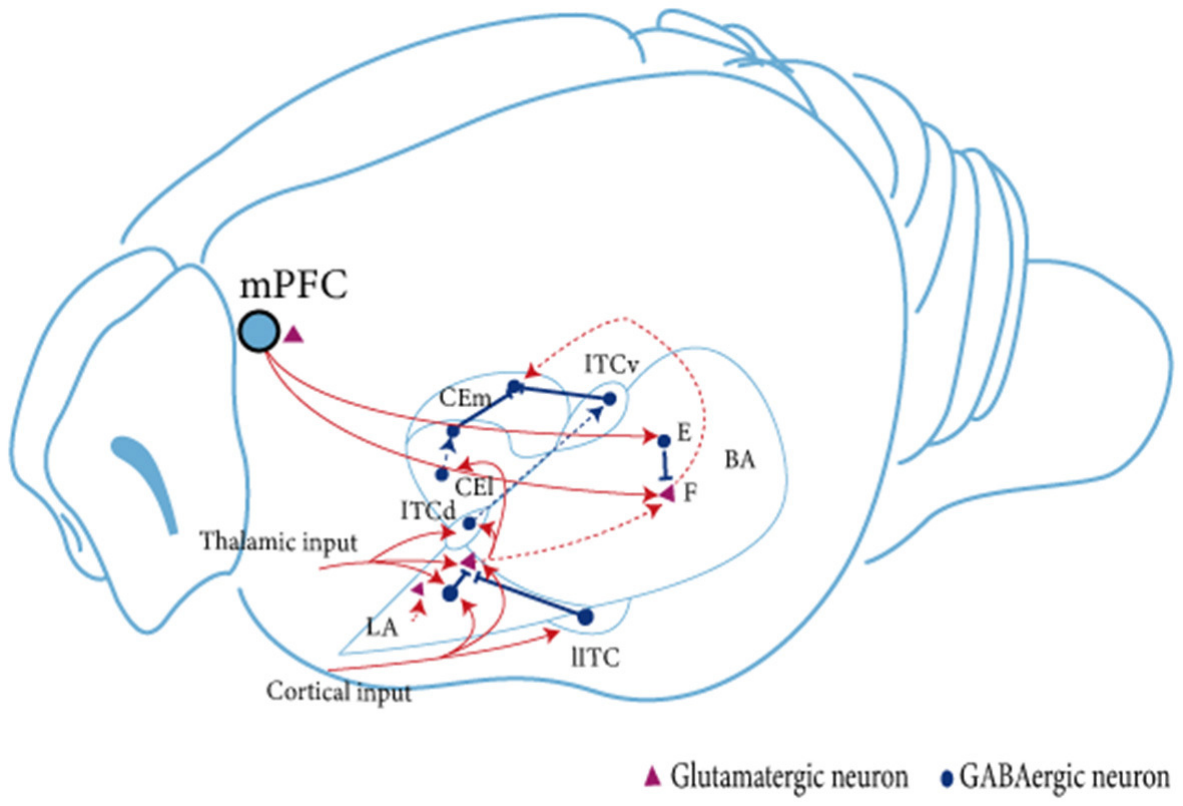
Schematic diagram of the linkage between amygdala subregions during fear extinction. The BLA receives signaling from the mPFC. Subsequently, in the BLA, neural signals are transmitted from dorsal to ventral, from LA to BA. The BLA projects unidirectionally to the CEA. LA projects mainly to CEl, whereas BA projects mainly to CEm. Neuron in the BLA affects neurons in the CEA brain region in two ways: directly through glutamatergic projections, or indirectly by stimulating ITC cells to produce feedforward inhibition in CEA neurons (Satish and Nair, 2012; Lee et al., 2013, 2017). Two types of glutamatergic projection neurons are present in the amygdala. BA, fear neuron and extinction neuron (Herry et al., 2008). The fear neuron is active in response to CS after fear acquisition and may mediate fear expression, whereas the extinction neuron is active in response to CS after fear memory extinction and may mediate fear inhibition; F, fear neuron; E, extinction neuron; IL, infralimbic cortex; BA, basal amygdala; LA, lateral amygdala; ITC, intercalated cell; lITC, lateral ITC; ITCd, dorsal ITC; ITCv, ventral ITC; CEm, medial CEA; CEl, lateral CEA.

The AMY is a central brain region involved in regulating fear memory. It is mainly divided into the BLA, the CeA, and the GABAergic intercalated cells (ITCs; see Figure 1; Capogna, 2014; Marek and Sah, 2018). The BLA can be further divided into the lateral amygdala (LA), which is the main site of sensory input to the AMY complex and receives substantial input from the sensory cortex and thalamus, and the basal amygdala (BA), which receives less direct sensory information but is interconnected with other brain regions such as the prefrontal cortex or ventral hippocampus (vHPC; Dejean et al., 2015). In addition, the BLA sends projections to the CeA, the main output area of the AMY, which contains GABAergic intermediate spiny neurons (Mcdonald, 1982); CeA mediates motor and autonomic responses to fear and stress by targeting the midbrain and hypothalamus (Krettek and Price, 1978). The lateral clusters of ITCs provide feedforward inhibition of BLA, while the medial clusters mainly block interactions at the interface between BLA and CeA (Asede et al., 2015). The different clusters of ITCs antagonize each other via inhibitory synapses and act differently on cortical and midbrain projection AMY output pathways to regulate fear state switching (Hagihara et al., 2021).

The BLA is a critical area for conditioned fear extinction (Davis, 1992; Krabbe et al., 2017). Inhibition of BLA activity before fear extinction training impairs the acquisition of extinction memory, whereas impairment of ITCs after fear extinction training inhibits the extinction of conditioned fear (Likhtik et al., 2008). The BLA contains mainly 80% glutamatergic principal neurons and 20% GABAergic interneurons (McDonald, 1982; McDonald and Augustine, 1993). The interaction of excitatory glutamatergic principal neurons and inhibitory interneurons may mediate the involvement of the AMY and regulate the fear extinction process. Evidence suggests that the consolidation of extinction memory depends on the interaction between BLA and IL, caused by the projection of principal neurons between brain regions (Maeng et al., 2017). During fear extinction, excitatory neurotransmitters are delivered to the BLA via the IL and activate ITCs (Berretta et al., 2005; Jüngling et al., 2008). During this process, projections are not reduced as ITC neurons are inhibitory. Thus, both excitation and inhibition play an equally important role (Cho et al., 2013). Studies have shown three types of principal neurons in the BA region of the AMY: fear cells, extinction cells, and extinction-resistant cells (Herry et al., 2008). Senn et al. found that inhibition of the BA–PL circuit during the fear extinction phase reduced the fear response in mice, while inhibition of the BA–IL circuit produced the opposite effect (Senn et al., 2014). In addition, fear cells in the BA project mainly to the PL, and the excitability of the cells is significantly increased during fear expression. In contrast, extinction-resistant cells project mainly to the IL and participate in the fear extinction process. It has also been reported that all BLA principal neurons consist of two genetically, functionally, and anatomically distinct neuronal populations (Kim et al., 2016, 2017). Among these, the R-spondin 2-expressing (Rspo2^+^) neurons are located in the anterior BLA (aBLA) and the protein phosphatase 1-regulatory inhibitor subunit 1B-expressing (Ppp1r1b^+^) neurons are located in the posterior BLA (pBLA), wherein the latter's optogenetic activation encourages the extinction of fear (Zhang et al., 2020). PKCδ neurons in the lateral part of the central amygdala (CEL) play an essential role in emotional learning by controlling local or downstream neuronal synaptic plasticity and integrating different sensory modalities, valence, and attentional signals (Whittle et al., 2021).

In contrast to excitatory principal neurons, GABAergic interneurons have thin, spiny dendrites with axons that are usually restricted to the BLA (Kemppainen and Pitknen, 2000). A small proportion of BLA GABAergic interneurons of unknown function have remote projections to regions including the basal forebrain or internal olfactory cortex (Kemppainen and Pitknen, 2000; Bienvenu et al., 2015). Although GABAergic interneurons occupy a relatively small fraction of the whole BLA, GABAergic neurons still play a crucial role in fear extinction. GABA can be formed by at least two glutamate decarboxylases (GAD), including GAD65 and GAD67 (Likhtik et al., 2008). Based on associative learning theory, fear generalization is an increase in the strength of the fear correlation (Kaczkurkin et al., 2017), while fear extinction is not a complete diminution or disappearance (Vervliet and Geens, 2014). It was shown that GAD65^−/−^ mice exhibited enhanced fear generalization (Sangha et al., 2009; Müller et al., 2015). During fear generalization, hippocampal and AMY theta frequencies exhibited an increase in synchronization (Sangha et al., 2009). Impairment of cued fear extinction was observed in GAD65^−/−^ mice (Sangha et al., 2009). Mounting data have recently highlighted the importance of GABAergic inhibitory interneurons during fear extinction (Bauer and LeDoux, 2004; Szinyei et al., 2007). GABAergic interneurons play an essential role through cell types and even subcellular compartments. GABAergic interneurons dynamically regulate neuronal excitation in a cell- and even subcellular-specific manner, exerting precise temporal control over activity in neuronal circuits (Krabbe et al., 2017; Cardin, 2018). However, little has been reported on the mechanisms of how inhibitory neurons within the BLA dynamically control and coordinate the neural circuits of fear extinction.

The BLA contains a variety of interneurons whose marker expression, basic properties, and connectivity are very similar to those of neocortical circuits. Based on early immunohistochemical studies, two major, non-overlapping groups of BLA interneurons can be defined by Calbindin protein (CB) and Calretinin protein (CR) expression. CB^+^ interneurons account for ~60% of BLA interneurons and can be further subdivided into PV, SST, neuropeptide Y (NPY), and cholecystokinin (CCK)-expressing cells (McDonald and Mascagni, 2002). Approximately 20% of BLA interneurons are CR^+^, many expressing CCK or VIP alone or partially overlapping (Mascagni and McDonald, 2003). A substantial overlap in the expression of SST and NPY has been reported, while the other categories are largely independent (Vogel et al., 2016). In BLA, SST^+^ interneurons are part of the inhibitory circuit, and they are usually targeted and regulated by the activity of PV^+^ and VIP^+^ interneurons (Krabbe et al., 2019). PV^+^ interneurons are one of the major subclasses of interneurons in the BLA (Atallah et al., 2012). PV^+^ interneurons preferentially form synapses in the peripheral regions of their target cells, thereby controlling neuronal activity (Basco et al., 2010). PV^+^ interneurons can form synaptic connections with other PV^+^ interneurons, glutamatergic interneurons, and GABAergic interneurons (Woodruff and Sah, 2007b). For example, during fear extinction, the number of activated GABAergic interneurons in the BLA increased significantly, and the synaptic terminals of PV^+^ interneurons around silent BA principal neurons increased (Li and Mcnally, 2014); damage to axon–axon inhibitory synapses in the BLA impairs fear extinction (Tsvetkov et al., 2002). It was shown that during fear conditioning, PV^+^ interneurons in the BLA responded differently to CS and US stimuli: 80% of PV^+^ interneurons showed increased activity during US stimulation; 75% of PV^+^ interneurons responded to CS-associated US stimulation, and 50% responded to independent US; while in CS-responsive PV^+^ interneurons, about 2/3 had enhanced activity and the rest had reduced activity (Krabbe et al., 2019). Another study used optogenetic manipulation to reveal that PV^+^ interneurons and SST^+^ interneurons within the BLA control fear behavior bidirectionally through two different de-inhibitory mechanisms in conditioned and unconditioned stimuli, respectively. The inhibitory/de-inhibitory microcircuits formed by both interneurons have an essential and unique role in associative learning (Wolff et al., 2014). However, the role of inhibitory neural microcircuits within the BLA in fear extinction remains to be investigated in depth.

#### 4.1.3. Hippocampus

The HPC is a crucial structure for processing contextual information and comprises four subregions: the DG and CA1–CA3. Cajal first noticed the difference in HPC across the dorsoventral axis and divided the HPC into the dorsal hippocampus (dHPC) and vHPC in 1901. The HPC is regionally specific in function, with different regions playing different roles in cognition and emotion (Zuo et al., 2022). There is substantial evidence that the dHPC is involved in spatial memory, while the vHPC is strongly associated with stress and emotion (Fanselow and Dong, 2010). For decades, a growing number of studies have emphasized the critical role of the HPC in the process of fear extinction. For example, the injection of DCS into the HPC facilitates fear extinction by promoting the differentiation of newly born cells (Ren et al., 2013). When administered systemically during contextual fear extinction, nicotine injections increased c-Fos expression in vHPC without affecting expression in dHPC. Similarly, injecting nicotine into vHPC but not dHPC promoted the extinction of contextual fear memory and showed a downregulation of GABA synthase GAD65 and GAD67 protein levels in vHPC (Kutlu et al., 2018). Recently, Umemori et al. used optogenetic techniques to achieve temporal and spatial control of neuronal plasticity *in vivo* and increased plasticity of pyramidal neurons by blue light excitation of photoinducible TrkB. Results further supported that the plasticity of pyramidal neurons in vHPC is a crucial mechanism for processing contextual fear memory extinction (Umemori et al., 2021).

Considering that the HPC is more strongly involved in contextual fear memory, which is closer to the clinical situation of PTSD, we mainly suggest more evidence about the HPC and contextual fear memory extinction. For instance, dHPC mediates the context-specific expression of fear memory (Hobin et al., 2006; Ji and Maren, 2007). Generally, after extinction training, rats showed lower freezing in the extinction situation and higher freezing outside the extinction situation. A study found that reversible inactivation of dHPC before extinction training significantly inhibited but did not completely block the acquisition of fear extinction memory in rats, showing a reduction in freezing (Corcoran et al., 2005). The reversible inactivation of dHPC during extinction training can disturb the situational coding of extinction memory, which is manifested by the impairment of fear extinction memory expression and the increase of freezing in rats no matter whether the extinction training occurs in the original conditioned situation or the second context (Corcoran et al., 2005). This suggests that dHPC is involved in the expression of context-dependent extinction memory and disrupts the renewal of fear extinction memory. Recently, Ressler et al. used chemical genetics techniques combined with a backward conditioning procedure suggesting that inhibition of protein synthesis within HPCs indirectly impaired the reconsolidation of contextual fear memories and clarified the possible mechanism behind the hippocampal regulation of contextual fear memory (Ressler et al., 2021).

It is worth mentioning that the vHPC forms projection relationships with other regions (including BLA, mPFC, hypothalamus, and vomeronasal nucleus) to form neural circuits for emotion regulation. In particular, the vHPC–mPFC neural circuit is involved in the extinction process of fear memory. Among them, BDNF signaling in the vHPC–IL circuit plays a decisive role in the cued fear extinction process (Rosas-Vidal et al., 2014). For example, 30 min after injecting BNDF into vHPC, the firing rate of more than half of IL neurons increased significantly and PL neurons decreased significantly (Rosas-Vidal et al., 2014). Inactivation of the circuit between vHPC and IL (in which proBDNF plays a mediating role) also impairs the acquisition of fear extinction, showing impairment of synchronous circadian rhythms of proBDNF and abnormalities in the basal firing rate of neurons. Furthermore, specific signaling pathways (e.g., BDNF/TrkB) can similarly regulate protein synthesis in the mPFC, thereby affecting fear extinction. Thus, BDNF in vHPC may promote fear memory extinction by exciting IL neurons (Rosas-Vidal et al., 2014). The BDNF and its prodomain (proBDNF) may be the key molecular mediators of the HPC–IL circuit (Peters et al., 2010). Furthermore, injection of the BDNF methionine (MET) prodomain in HPC neurons leads to a breakdown of dendritic spine density, altering the projections to PL and subsequently impairing the neural circuitry for fear memory extinction. Specifically, after extinction training, adolescent knockout mice (BdnfMet/Met) exhibit impaired fear extinction as evidenced by higher levels of freezing, lower vCA1–PL projection connectivity, and abnormal firing rates of vCA1 neurons during fear extinction compared to the adolescent group (BdnfVal/val). More direct evidence suggested that manipulating the activation of vCA1 projections to PL inhibitory interneurons can modulate fear memory expression during extinction training (Sun et al., 2019b).

**Figure 2:**
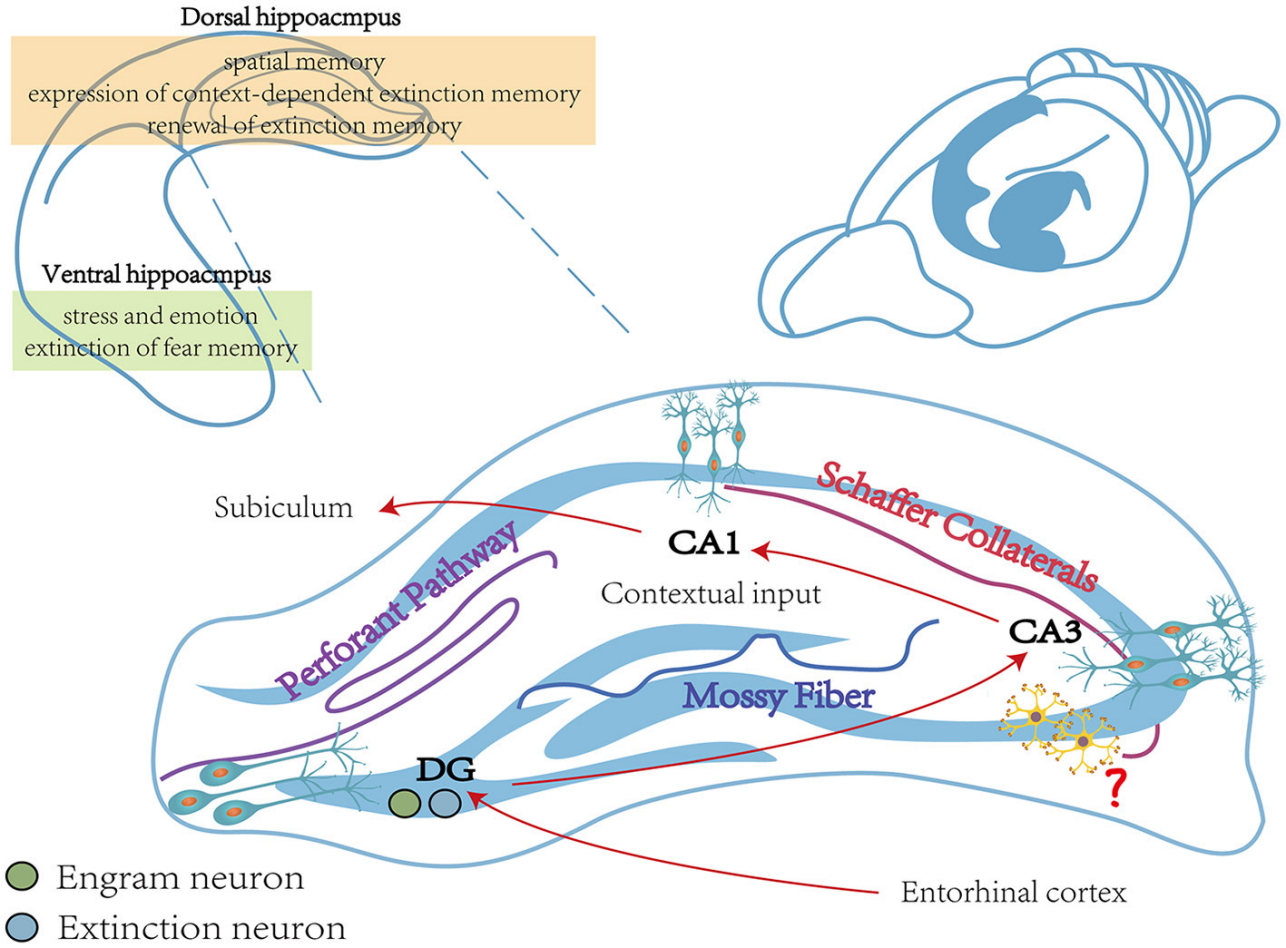
The main function of the hippocampus during fear memory. The vHPC is closely linked to stress and emotion and is involved in the extinction process of fear memory (Rosas-Vidal et al., 2014). The dHPC is engaged in spatial memory and involved in the expression of context-dependent extinction memory and the renewal of fear extinction memory (Corcoran et al., 2005). The vHPC includes three different pathways, including the Perforant pathway, the Mossy Fiber pathway, and the Schaffer Collaterals pathway, which together consist of a trisynapic circuit (Catlow et al., 2016). Contextual memory-specific representations are supported by trisynaptic circuits from the cortex to the DG and from CA3 to CA1. Cortical perceptual input and memory-extracted projections from CA3 are both received by CA1, and it is also required for contextual inputs (Schapiro et al., 2017; Sans Dublanc et al., 2020). The role of interneurons in the hippocampus remains to be explored (Wittner et al., 2006). vHPC, ventral hippocampus; dHPC, dorsal hippocampus.

In addition, the HPC includes different long-term depression (LTD)-induced pathways, such as the HPC perforant pathway, the mossy fiber pathway, and the Schaffer collaterals pathway (see Figure 2; Catlow et al., 2016). Among them, the HPC perforant pathway refers to transmitting signals from the DG via granule cell fibers in the entorhinal cortex (Kajiwara et al., 2008). The mossy fiber pathway projects dentate granule cells to pyramidal cells in the CA3 region via mossy fibers. The Schaffer collaterals branch pathway refers to the signal that CA1 pyramidal neurons receive from the Schaffer collaterals of the axons of CA3 pyramidal neurons (Park et al., 2022). It was shown that rats constructed by the UWT model exhibited impaired fear extinction. Compared to wild-type rats, LTD of the Schaffer collaterals branch pathway and the HPC perforant pathway were significantly reduced in the vHPC and dHPC of UWT rats. Therefore, impaired LTD of the HPC pathway may be necessary for impaired fear extinction (Zuo et al., 2022).

DG engram cells are critically involved in regulating fear expression (Lacagnina et al., 2019). During this process, a sparse ensemble of DG granule cells, called “fear engram cells”, is activated (Denny et al., 2014; Lacagnina et al., 2019). In addition to the BLA, “extinction neurons” also exist in DG (Herry et al., 2008; Lacagnina et al., 2019). Evidence showed that ArcCreER^T2^ transgenic mice exhibited reduced activation of “fear engram cells” and increased activation of “extinction neurons” in DG during the test session 5 days after extinction training, while “fear engram cells” were activated and “extinction neurons” were suppressed during the spontaneous recovery test 28 days after extinction training (Lacagnina et al., 2019). It was also shown that by silencing neurons labeled in the DG during extinction, transgenic mice exhibited more freezing after extinction training. The finding of extinction neurons reflects the collection representations in HPC and suggests that the extinction of fear memory may depend on the competition between these collection representations.

In addition, the study suggests that changes in gamma wave power and synchronization between brain regions are essential mechanisms for regulating fear emotions (Courtin et al., 2014). The network of inhibitory interneurons, consisting of fast-firing, PV-expressing basket cells, is the main force in generating gamma neural oscillations (Lasztóczi and Klausberger, 2014). However, no evidence has been retrieved to support the direct involvement of gamma wave oscillations or PV^+^ interneuron activity in the HPC in the fear extinction process. In addition, a set of PV^+^ interneurons in the HPC regulates the output of the interneurons together with the SST^+^ interneurons.

**Figure 3:**
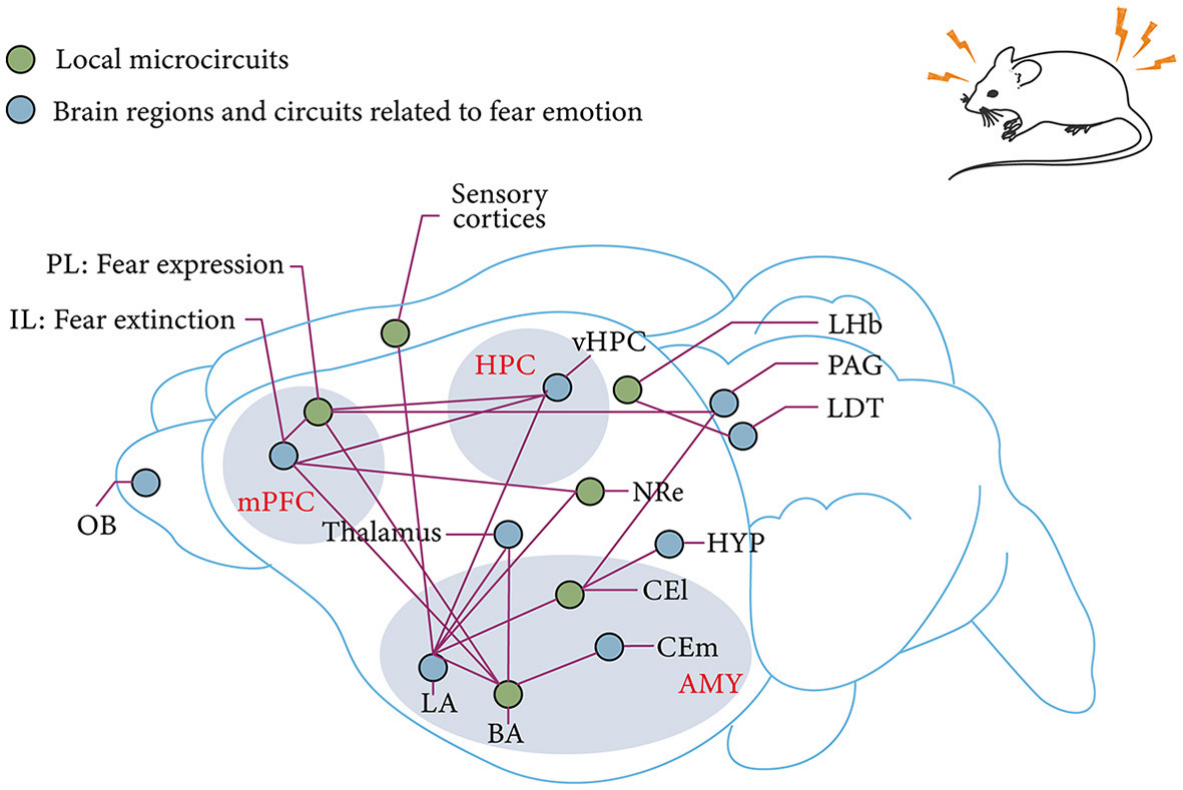
mPFC–AMY–HPC tripartite brain network associated with fear and fear extinction. IL and PL in the mPFC are associated with fear extinction and fear expression, respectively (Sierra-Mercado et al., 2011; Giustino and Maren, 2015). Subnuclei of the AMY, which receive sensory input from the thalamus in the cortex, are also the main site of fear memory plasticity (LeDoux, 2003; Olmos-Serrano and Corbin, 2011). This plasticity receives modulation from the reciprocal projections of BLA and vHPC as well as the reciprocal projections of BLA and PL (Felix-Ortiz et al., 2013; McGarry and Carter, 2017). The extinction of fear memory relies on a similar circuit as the establishment of fear memory. The pathway from IL to BLA is derived from the fear output of CEl to HYP and PAG (Tovote et al., 2015). Thalamic–AMY circuits are involved in remote fear memory extinction (Silva et al., 2021). The LHb and the LDT are involved in innate fear memory (Pereira and Moita, 2016). mPFC, medial prefrontal cortex; HPC, hippocampus; AMY, amygdala; OB, olfactory bulb; IL, prelimbic cortex; PL, prelimbic cortex; BLA, basolateral amygdala; BA, basal amygdala; LA, lateral amygdala; vHPC, ventral hippocampus; CEm, medial central amygdala; CEl, lateral central amygdala; HYP, hypothalamus; NRe, thalamic nucleus reuniens; LDT, laterodorsal tegmentum; PAG, Periaqueductal gray; LHb, lateral habenula.

Above all, conditioned fear extinction requires the joint involvement of mPFC, BLA, and HPC and forms a dynamic neural network. However, despite the advances in optogenetics, chemical genetics, and viral genetic engineering, research on the mechanisms surrounding fear extinction has gradually transitioned from the previous non-real-time, single-brain region or brain-to-brain neural circuits and molecular mechanism studies to the exploration of the mechanisms of action and their functional associations among more refined subregions and even subcellular populations in brain regions (Figure 3). However, local neural microcircuit mechanisms targeting key brain regions for fear extinction remain to be explored.

### 4.2. Molecular mechanisms of fear extinction

There have been numerous studies of fear extinction, and some progress in molecular mechanisms has been made, such as excitatory neurotransmitters (e.g., glutamate) or inhibitory neurotransmitters (e.g., aminobutyric acid) and their receptors, neurotrophic factor (NTF) families and their receptors, related ion channels, and epigenetic modifications. An overview is provided below.

#### 4.2.1. Glutamate and its receptors

Glutamate is the main excitatory neurotransmitter in the brain (Schousboe, 1981; Sundaram et al., 2012), which opens ion channels on the cell surface by binding to its receptors (Sontheimer et al., 1988), and in turn induces electrical signals that lead to neuronal firing (Coutinho and Knöpfel, 2002). Glutamate is usually divided into ionotropic glutamate receptors (iGluRs) and metabotropic glutamate receptors (mGluRs; Nakanishi, 1992; Hollmann and Heinemann, 1994). Based on their different affinities for agonists, iGluRs are classified as NMDARs, α-amino-3-hydroxy-5-methyl-4-oxazolepropionic acid (AMPARs), and kainate receptors (KARs; Karakas et al., 2015). Among them, AMPARs mediate rapid excitatory synaptic transmission in the central nervous system (CNS) and are involved in long-term potentiation (LTP) and LTD (Heine et al., 2008). KARs are similar to AMPARs, except that there are fewer of them (Burnashev et al., 1996). mGLuRs contain a train of receptors. As G protein-coupled receptors, they can work through a second messenger system (Chałupnik and Szymańska, 2023).

Fear extinction is a new memory association process that requires enhanced synaptic plasticity. Therefore, administration of iGluRs agonists (e.g., NMDARs agonists) may excite target cells, leading to membrane depolarization and calcium inward flow, while releasing antagonists may inhibit the extinction process (Davis and Myers, 2002). For example, intra-amygdala injection of the NMDARs agonist, DCS, enhances extinction learning (Walker et al., 2002; Guercio and Panizzutti, 2018). In contrast, intra-amygdala injection of the NMDARs antagonist, AP5, before extinction training resulted in a dose-dependent attenuation of fear extinction (Falls et al., 1992). Intra-mPFC injection of ketamine (an antagonist of NMDARs) promotes fear extinction via mTOCR1 signaling (Girgenti et al., 2017). In addition, local intra-cerebroventricular injections of AICP, a novel glycine site agonist, can activate GluN2C-containing NMDARs to enhance fear memory extinction (Shelkar et al., 2021). In addition, NMDARs containing NR2A are necessary for fear memory extraction, but NMDARs containing NR2B only play a role in retrieving remote fear memory (Corcoran et al., 2015; Shi et al., 2018). This is closely related to the NR2B/PKA signaling pathway. Corcoran et al. used a variant of the FS model to monitor the changes in fear extinction. The study focused on the behavioral differences and molecular mechanisms of recent and remote contextual fear memory recovery in mice (Corcoran et al., 2013). It explored the mechanisms of the NR2B/PKA pathway in the Retrosplenial Cortex (RSC) by modulating changes in NMDARs and their related signaling pathways in the RSC through pharmacological techniques. The results showed that after memory recovery, the extinction process of remote fear memory was faster than that of recent fear memory, showing less freezing behavior (Shi et al., 2018). In addition, remote fear memory extinction depends on the signaling cascade processes involving NR2B activation, dissolution of NR2B/PKA complexes, reduced PKA activation, and reduced CREB phosphorylation in the RSC (Shi et al., 2018).

AMPARs are mainly constructed with two subunits, GluA1 and GluA2. GluA1 may relate to the fear memory expression (Clem and Huganir, 2010), while GluA2 is critical for fear extinction. Mice exhibiting higher GluA2 levels in the vmPFC also showed higher levels of fear extinction (Gourley et al., 2009). GluA2 is involved in the endocytosis of AMPARs (Ahmadian et al., 2004). Tat-GluA2 3Y is a synthetic peptide that prevents the expression of various forms of LTD by interfering with AMPAR-promoting endocytosis without affecting basal synaptic transmission or LTP. Administration of Tat-GluA2 3Y (3.0 μmol/kg) significantly inhibited the acquisition and retention of fear extinction memory, supporting the critical role of AMPARs (Bai et al., 2014). Another study also found that administration of Tat-GluA2 3Y during the formation or testing phase of fear conditioning did not affect fear memory expression or recall, and only administration 60 min before extinction training disrupted fear memory extinction (Dalton et al., 2008). From the perspective of molecular mechanism, the endocytosis process of AMPAR and the maintenance process of LTP all involve the role of Arc (Trent et al., 2017). Arc can change synaptic plasticity by controlling actin cytoskeleton rearrangements and then coordinating the consolidation process of extinction memories (Korb and Finkbeiner, 2011). Future research should focus more on the critical cellular and molecular mechanisms of glutamate receptors in mediating fear extinction to develop targeted drugs better.

Administration of D-Serine facilitated the extinction of fear memory by blocking the endocytosis of AMPARs (Labrie et al., 2009; Bai et al., 2014). PEPA, an AMPA receptor enhancer, plays the same role as D-Serine (Zushida et al., 2007; Yamada et al., 2011). A dose of 30 mg/kg, but not 3 mg/kg or 10 mg/kg of PEPA, can significantly promote the extinction of fear memory in mice (Zushida et al., 2007). The researchers suggested that the compensation for the weakening of mPFC stress activation may mediate the promotion mechanism of PEAP on fear memory extinction. Thus, the effect of the PEPA is dose-dependent. It is necessary to clarify the safe and effective dose concentration range of enhancers in the future. Moreover, focusing on the effective time window for fear extinction makes the drug work better. For instance, the reconsolidation process of memory means that the long-term memory that has been consolidated will go through a volatile and sensitive stage after activation and then stabilize again. The original memory can be modified, strengthened, changed, or even eliminated during this process. Mastering this process is critical to erasing the fear memory. The initial phase of synaptic depression, 1–4 h after fear memory formation, is the time window in which memory returns to an unstable state, which is essential for reconsolidation. Evidence showed that no significant effect of disruption of GluA2 surface expression in the HPC after 1 day might relate to the time window of memory (Nader et al., 2000; Serrano et al., 2008; Migues et al., 2010).

It is generally accepted that inhibition of the activity of the glutamatergic system impairs the extinction of fear memories. However, both systemic and local BLA injections of riluzole enhanced the fear of extinction (Sugiyama et al., 2018), which has been shown to inhibit glutamatergic transmission (Benoit and Escande, 1991; Kretschmer et al., 1998). It is speculated that this may be because (1) riluzole will increase the cell surface expression and transmission of AMPA subunits GluR1 and GluR2, thereby affecting the regulation of AMPAR (Du et al., 2007); (2) the enhancing effect of riluzole on fear extinction may be indirectly influenced by the anxiolytic-like effect of riluzole when BLA is administered (Sugiyama et al., 2018).

From a pharmacological point of view, KARs and AMPARs share several agonists and antagonists, so they are very similar, and it is not easy to make a clear distinction. Previous reviews often discuss the two, collectively referred to as non-NMDA receptors (Harvey and Shahid, 2012; Niciu et al., 2012). However, AMPAR-specific antagonism by 2,3-benzodiazepines, particularly GYKI 53655 (LY300268), provides the basis for distinguishing the roles of AMPARs and KARs (Paternain et al., 1995; Wilding and Huettner, 1995). KARs are present in the peripheral nervous system (PNS), spinal cord, AMY, and HPC of the CNS (Rydgren, 2018; Sargin, 2019; Mennesson et al., 2020). KARs are essential in sensory transmission, pain inflammatory response, fear memory, and learning and memory partly because they play a role in synaptic plasticity in the AMY and HPC, which is closely related to LTP (Ko et al., 2005; Bhangoo and Swanson, 2013). Evidence suggests that GluR6, one of the KAR subunits, plays an important role in fear memory. GluR6 knockout mice showed a significant decrease in the freezing on the 1st day, the 3rd day, the 1st week, and the 2nd week after the establishment of fear conditioning, that is, the significant reduction of situational fear memory and auditory fear memory reduced (Ko et al., 2005). Further results showed that GluR6-knockout mice exhibited LTP blockade and synaptic potentiation (pairing synaptic activity with postsynaptic depolarization) compared with wild-type mice or GluR5-knockout mice (Ko et al., 2005), which further reveal the mechanism of action of GluR6 in fear memory extinction.

The study found that NETO2 protein levels and surface expression were lower in GluR6-deficient mice (Zhang et al., 2009). NETO1 and NETO2 are auxiliary proteins for KARs. Among these, NETO2 is essential for fear extinction (Rydgren, 2018; Sargin, 2019). Studies showed that NETO2^−/−^ mice, which presented higher fear expression and lower fear extinction, can be a hopeful candidate for a PTSD-like model. Compared with NETO2^+/+^ mice, NETO2^−/−^ mice exhibited immaturity and hyperactivity of the amygdala, which corresponded with the prolonged extinction process (Mennesson et al., 2020). However, this cannot match the enhancement of glutamatergic synapses. In addition, NETO2^−/−^ mice showed an induced abundance of synapses of the KARs subunits in fear-related circuits, meaning that NETO2 may play a critical role in forming synapses during fear extinction (Sargin, 2019). This relationship was also supported by measurements of the number of c-Fos positive cells, delivery of glutamatergic and GABAergic systems, and spine density of thin dendrites (Mennesson et al., 2020).

The mGluRs can inhibit or promote intrinsic neuronal excitability and alter the plasticity of synaptic structures, which is also essential for fear extinction. The de-enhancement effect refers to the significant increase in thalamic-LA synaptic efficacy after auditory cue fear acquisition and reversing the increased thalamic-LA synaptic efficacy by fear extinction (Hong et al., 2009). mGluR1 activity may be a potential molecular mechanism for the de-enhancement effect, mainly mediated by the endocytosis of AMPAR (Hong et al., 2009). In addition, the de-enhancing effect of auditory cortex-LA synapses is also closely associated with fear extinction, which is regulated by NMDARs and mGluR2 (Hong et al., 2009). It was also found that administration of mGlu3 negative allosteric modulators impaired the mPFC-dependent fear extinction process. 129S1/SvImJ (S1) inbred mice showed severe impairment in fear extinction (Hefner et al., 2008), especially the mPFC–BLA circuit-mediated fear extinction process (Herry et al., 2010). However, a Zn-restricted diet (ZnR) can alleviate the impaired extinction in S1 mice without affecting fear acquisition or expression. This may be because ZnR increases activation in brain regions associated with fear extinction and decreases activation in brain regions associated with fear acquisition or expression and compared with C57BL/6J strain mice, S1 mice had poorer fear extinction with increased levels of mGluR7 mRNA in the BLA region (Whittle et al., 2010). However, the current exploration of the role of mGluRs in fear extinction has almost always used unconditional knockdown of specific genes or gene silencing techniques, and various technical tools need to be developed in future to elucidate the mechanism of the role of mGluRs in fear extinction.

#### 4.2.2. γ-aminobutyric acid and its receptors

According to their distribution areas, GABAergic neurons in the AMY can be roughly divided into local GABAergic interneurons scattered around the BLA and the lateral parascapular cells (LPCs; Jasnow et al., 2009; Silberman et al., 2010). The GABAergic intercalated cell clusters contribute more to the fear extinction process. For example, endogenous neuropeptide S (NPS) can regulate the extinction process by modulating transmission from glutamatergic to GABAergic neurons, and mRNA for NPS receptors is widely present in the intercalated cell mass. Injection of the NPS receptor antagonist SHA 68 into LA/BLA 2 h before extinction recall that it impairs the extinction process of fear memory in mice, as evidenced by higher freezing levels throughout the extinction training (Jüngling et al., 2008). Skelly et al. observed inhibition of extinction learning 24 h after extinction training by injecting adrenergic receptors (β3-Ars) into the BLA 24 h before extinction training in adult male rats (Skelly et al., 2016). β3-ARs may enhance the inhibitory synaptic plasticity of GABAergic neurons in lateral parietal cells *in vivo* and inhibit the activity of BLA pyramidal neurons. GABAergic interneurons can coordinate the activity of principal excitatory cells with low resting firing rates (Woodruff and Sah, 2007a; Basco et al., 2010). For example, neurofascin, present in the axon initial segment (AIS) of BLA projection neurons, is responsible for initiating action potentials (Zonta et al., 2011). By knocking out neurofascin in BLA, inhibitory synapses of AIS can be specifically removed, meaning that a lack of neurofascin can reduce GABAergic input to inhibit abnormal excitatory principal neurons and thus further disrupt the fear extinction process. Saga et al. observed that synapses in BLA were impaired and that the neurofascin-knockdown mice reduced the postsynaptic function of GABAergic synaptic transmission in neurofascin-knockdown mice (Saha et al., 2017). Behavioral findings suggested that fear extinction processes are damaged, but fear expression and acquisition are not. Nicotine injection also downregulates GAD65 and GAD67 in vHPC, inhibiting GABAergic neuronal activity and impairing contextual fear memory extinction.

#### 4.2.3. Cholinergic neurotransmitters and their receptors

The cholinergic nervous system is distributed in almost all regions of the brain. There are two major cholinergic projection systems in the brain, including the magnocellular basal forebrain (BF) cholinergic system and the brainstem cholinergic system (Xiao et al., 2016; Merino, 2019). The former consists of the nucleus basalis meynert (NBM), the medial septum (MS) nucleus, and the horizontal, diagonal band of Broca (HDB), which project extensively to areas such as the cortex and HPC; the latter projects from the teg-mental peduncle to the thalamus and midbrain (Nagasaka et al., 2017). Although the number of cholinergic neurons in each region is not large, they regulate numerous behaviors with their extremely sparse distribution. The NBM projections include the neocortex and BLA, critical fear-expression regions (Knox, 2016). Thus, different groups of cholinergic neurons may mediate the extinction of fear memory.

In rodents, ILs receive projections from the NBM. Using whole-cell patch clamp recordings, Santini et al. found that the intrinsic excitability of IL neurons incubated in muscarine chloride (10 uM) was enhanced by decreased M current and slow post-hyperpolarization (Santini et al., 2012). After muscarine application, the addition of the XE-991 (an M-type K^+^ channel antagonist, 10 μM) did not cause a further increase in the number of evoked spikes (Santini et al., 2012). Subsequently, the researchers used auditory FC to examine the role of muscarinic receptors in fear extinction. Results showed that rats injected systemically with scopolamine (a muscarinic receptor antagonist) before or after extinction training exhibited lower freezing levels after 24 h than controls (Santini et al., 2012). This implies that cholinergic input to IL may play a role in regulating fear extinction. Cholinergic input from the BF to the neocortex and HPC is also critical for fear memory extinction. Tronson and colleagues used transgenic cFos-LacZ to explore the activation of hippocampal CA1 neurons during FC and extinction (Tronson et al., 2009). Immunotoxin, a combination of saporin and rabbit anti-mouse neurotrophin low-affinity receptor antibodies, induced lesions in MS neurons and subsequently led to cholinergic input to the HPC. When the cholinergic output from the MS to the HPC was manipulated by immunotoxin injection, results showed that cholinergic projections from the MS to the HPC selectively reduced the extinction of fear memory without affecting fear expression. Subsequently, Knox and Keller induced cholinergic lesions in areas of the MS/vDBB in response to the extinction of cued and contextual fear memory (Knox and Keller, 2016). The results showed that lesions in the cholinergic system induced fear memory generation and disrupted the generalization of contextual fear memory and the acquisition of cued fear extinction, during which rats exhibited higher freezing levels. This may imply that, besides the generally accepted fear extinction neural circuits described above, the cholinergic circuits constituted by MS/vDBB may also be involved in the fear memory extinction process. However, evidence also shows that BF cholinergic lesions do not affect contextual fear extinction (Frick et al., 2004). FC-induced stress enhancement may also change this process (Chang and Maren, 2009). Recently, Crimmins et al. further used techniques such as optogenetics and viral tracing to reveal the role of basal forebrain cholinergic signaling in the BLA in regulating the formation, extinction, and renewal of fear memory (Crimmins et al., 2023). It was shown that silencing ^Ach^NBM → BLA during fear conditioning enhanced the extinction of fear memory, whereas silencing ^Ach^HDB → BLA had no significant effect in both the extinction and recovery phases. They then used optogenetic silencing of ^Ach^NBM → BLA and ^Ach^HDB → BLA during fear extinction to show that silencing of ^Ach^NBM → BLA and ^Ach^HDB → BLA during extinction enhanced fear extinction, but only silencing of ^Ach^HDB → BLA prevented the updating of fear memory (Crimmins et al., 2023). In conclusion, the available evidence supports the role of the cholinergic system in fear extinction, especially the critical role of the two neural circuits ^Ach^NBM → BLA and ^Ach^HDB → BLA in fear extinction, which has important clinical implications for the treatment of stress-related disorders.

#### 4.2.4. Monoamine neurotransmitters and their receptors

Monoamine neurotransmitters include catecholamines and indoleamines, of which catecholamines are mainly composed of dopamine, norepinephrine, and epinephrine, and indoleamines are especially 5-hydroxytryptamine (5-HT; File, 1985; Bernardy and Friedman, 2015). The monoamine neurotransmitter system is involved in emotion, memory, and arousal processes and plays a vital role in the production of astrocytes and the secretion of NTF3. Most of the drugs used in trauma and fear-related psychological disorders [e.g., anxiety; e.g., selective serotonin reuptake inhibitors (SSRIs) and serotonin and noradrenaline reuptake inhibitors (SNRIs)] have effects on monoamine function (Bernardy and Friedman, 2015; MacNamara et al., 2016). Injection of 5-HT1A receptor agonists into hippocampal CA1 leads to the inhibition of neural activity by 5-HT1A receptors, mainly in inhibitory interneurons, and impairs the extinction of fear memory (Nachtigall et al., 2019). In contrast, applying the 5-HT3 antagonist ondansetron enhances extinction (Mohammadi-Farani et al., 2021). 5-HT3A knockout mice show impaired contextual and cued fear memory extinction (Mohammadi-Farani et al., 2021). 5-HT3 receptors may be involved in the extinction process by mediating GABAergic neurotransmission. Furthermore, novelty can inhibit extinction enhancement according to the synaptic tagging and capture (STC) hypothesis. Intra-hippocampal CA1 injections of β-adrenergic receptor antagonists inhibit the enhancing effects of this process (Nachtigall et al., 2019). This may be because β-adrenergic is closely related to LTP. Applying a β-adrenergic agonist (e.g., isoprenaline) can induce the shift in LTP (Huang and Kandel, 1996; Straube et al., 2003).

#### 4.2.5. Glucocorticoids and their receptors

There is substantial evidence that glucocorticoids promote fear extinction, but glucocorticoid receptor (GR) antagonists impair the extinction process (Perez et al., 2022; Raju et al., 2022). FK506-binding proteins 4 and 5 (FKBP4 and FKBP5) are two essential proteins in GR-dependent transcriptional activity (Lin et al., 2020). Studies suggest that FKBP5 may be a candidate gene for human PTSD treatment. FKBP5 regulates hypothalamic-pituitary-adrenal axis (HPA) activity and regulates stressful stimuli (Choi et al., 2021). When subjects encounter threatening cues or stressful triggers, the HPA axis is activated, and the adrenal glands release glucocorticoids (e.g., cortisol in humans or corticosterone in rodents). Subsequently, FKBP5 can bind competitively to GR during glucocorticoid and GR interactions, reducing its affinity for glucocorticoids. Thus, aberrant expression of FKBP5 may lead to maladaptive behaviors, such as PTSD-like symptoms. Evidence suggests that downregulating FKBP5 expression levels in IL mPFC may promote fear memory extinction (Criado-Marrero et al., 2017). Furthermore, impairment of the HPA axis may contribute to developing maladaptive behaviors such as PTSD-like symptoms. Additionally, damage to the HPA axis may be a potential cause of persistent fear memories in PTSD patients. Dexamethasone is a glucocorticoid receptor agonist, and systemic administration of dexamethasone inhibits the activity of the HPA axis (Sawamura et al., 2016). It has been shown that dexamethasone given before extinction training may enhance the extinction of fear memory by altering FKBP5-mediated glucocorticoid sensitivity, a process that may involve epigenetic modulation, including dynamic changes in the Dnmt and Tet gene pathways (Sawamura et al., 2016). However, dexamethasone exhibits a dose-dependent mechanism of action in fear extinction (Galatzer-Levy et al., 2017). Low doses of dexamethasone did not alter the mRNA expression levels of FKBP5 in the AMY or the performance of extinction.

#### 4.2.6. Endocannabinoid system

The relationship between the endocannabinoid system (ECS) and learning and memory associated with traumatic stress has gained widespread attention in the last decade. Surveys have shown that an increasing number of patients in the PTSD population are relieving their adverse symptoms by smoking cannabis (Kessler et al., 1995; Cornelius et al., 2010). Surveys in regions and states where cannabis use has been legalized in the United States have also shown that PTSD patients' symptoms are significantly relieved after cannabis use (Greer et al., 2014). Studies have shown that PTSD patients may be accompanied by dysfunction of the ECS, as evidenced by abnormal alterations in both endogenous cannabinoid levels and their receptors in the blood of PTSD patients (Hauer et al., 2013; Neumeister et al., 2013). The ECS is composed of endogenous cannabinoids (lipid retrograde neurotransmitters) and cannabinoid receptors that are widely distributed in the mammalian CNS and PNS (Lu and Mackie, 2016). Endogenous cannabinoids consist mainly of anandamide (AEA) and 2-arachidonoylglycerol (2-AG), both of which are mainly synthesized postsynaptically and act as retrograde first messengers that regulate neurotransmitter release by activating cannabinoid receptors located in the presynaptic membrane (Gaoni and Mechoulam, 1964; Devane et al., 1992; Mechoulam et al., 1995). Cannabinoid receptors mainly include Cannabinoidreceptor1 (CB1R) and Cannabinoidreceptor 2 (CB2R; Mackie, 2005; Ibarra-Lecue et al., 2018). CB1R is predominantly expressed in brain regions associated with affective disorders and is highly expressed in brain regions associated with the development of PTSD (Ibarra-Lecue et al., 2018). In addition, CB1R has been shown to reduce pain, inflammation, and motor control, and modulate perceptual, memory, and cognitive functions (Milligan et al., 2020; Vasincu et al., 2022). Marsicano et al. found that CB1R knockdown or administration of its blocker strongly inhibited the extinction of auditory conditioned fear. This may relate to a large release of endogenous cannabinoids in BLA which was detected during fear memory extinction (Marsicano et al., 2002). These findings show that ECS plays an important role in the regulation of fear memory, suggesting that it may be a new potential target for the treatment of stress-related disorders.

More studies have been conducted on the role of ECS in the consolidation, retrieval, and extinction of fear memory. It was found that the consolidation of fear memory was not affected in mice injected intraperitoneally with CB1R antagonists (Arenos et al., 2006), the retrieval of fear memory was enhanced (Niyuhire et al., 2007; Lemos et al., 2010; Lisboa et al., 2010), and the extinction of fear memory was impaired (Do Monte et al., 2013; Gunduz-Cinar et al., 2013). Intraperitoneal injection of WIN55,212-2 (WIN), a CB1R agonist, significantly enhances fear memory recovery, and low doses of WIN promote fear memory extinction (Ghasemi et al., 2017). By microinjecting CB1R agonists or antagonists into brain regions associated with fear memory, it was found that rats injected with the CB1R agonist WIN in the BLA exhibited an impairment of fear memory retrieval, with no effect on fear extinction. Injection of the CB1R antagonist AM251 enhanced fear memory retrieval without effects on other stages of fear memory; injection of AM251 into the mPFC attenuated fear memory consolidation and impaired fear memory extinction (Kuhnert et al., 2013). Injection of the CB1R agonist WIN into the HPC of rats impaired the retrieval of contextual fear memory (Atsak et al., 2012). Direct injection of AEA into the HPC of rats enhanced the extinction of contextual fear memory, but co-injection of equal doses of the CB1R antagonist AM251 hindered this enhanced extinction effect (De Oliveira Alvares et al., 2008). The above results strongly suggest that cannabinoid receptor activation plays an important role in promoting memory extinction.

#### 4.2.7. Oxytocin system

Oxytocin (OXT) is a neuropeptide produced in the hypothalamus that acts on the peripheral and central nervous systems, exerting a neurotransmitter-like effect (MacDonald and MacDonald, 2010). Magnocellular neurons mainly synthesize OXT in the paraventricular and supraoptic nuclei of the hypothalamus, which project to the posterior pituitary gland for release into the peripheral venous circulation. Parvocellular neurons in the paraventricular nucleus project OXT to different areas of the brain (Ross and Young, 2009; Striepens et al., 2011). The neurons in the hypothalamus also project OXT to the AMY, HPC, midbrain, and frontal lobe brain regions. OXT modulates the function of the HPA axis and the autonomic nervous system, thus regulating the acquisition and extinction of fear (Abramova et al., 2020). For example, OXT release from the dHPC was significantly increased during auditory fear extinction. Freezing levels were significantly lower in mice injected with the OXT antagonist atosiban than in controls and mice injected with OXT within the HPC (Bazaz et al., 2022). Oxytocin can also facilitate or inhibit the fear extinction process by affecting the activity of brain regions such as the AMY and mPFC (Lahoud and Maroun, 2013; Gunduz-Cinar et al., 2020).

#### 4.2.8. Neurotrophic factor family and its receptors

BDNF is a signaling protein in the NTF family that has been shown to regulate synaptic transmission and its plasticity. BDNF can bind at least two receptors, including TrkB and LNGFR (also known as P75). TrkB and P75 are highly expressed in the human cortex and HPC regions (Rodríguez-Tébar et al., 1992; Lippi et al., 2020). Among them, BDNF and its high-affinity receptor TrkB play a crucial role in fear extinction. Building on previous work, Yuana et al. constructed a new tool, CC1-EGFP, which disrupts explicitly axonal transport of TrkB and uses it as a specific inhibitor, aiming to explore the potential mechanisms by which BDNF regulates fear extinction, in particular, the function of presynaptic TrkB in BLA neurons (Li et al., 2017). The results showed that CC1-EGFP mice exhibited higher freezing levels during extinction than EGFP controls, suggesting that CC1-EGFP impaired the extinction process. In contrast, administering a TrkB agonist (selective 7,8-dihydroxyflavone) facilitated the extinction of fear memory (Yang et al., 2019).

There are several single nucleotide polymorphism (SNP) loci in the BDNF gene, of which rs6525 has been widely explored (Li et al., 2021). In the prodomain of BDNF, mutations at this site result in an amino acid switch: the valine (Val) at position 66 in the codon is replaced by a MET and is named Val66MET (Egan et al., 2003). This allows the construction of animal models that mimic polymorphisms of specific human phenotypes using knockout mice. For example, studies from rodents and humans have shown that MET carriers exhibit significantly impaired extinction learning ability. During extinction, MET allele carriers have lower vmPFC activity and higher AMY activity than non-MET allele carriers (Soliman et al., 2010). This implies that BDNF may play a specific role between vmPFC and AMY. Indeed, BDNF signaling in BLA–IL and HPC–IL circuits facilitates memory extinction (Xin et al., 2014). In conclusion, the functions of BDNF (e.g., inhibiting the formation of the TrkB axonal forward transport complex, inducing the release of neural signals, and transducing TrkB retrograde signals) are crucial for the extinction of fear memory.

Nerve growth factor (NGF), a protein secreted by neuronal target cells, is the prototypical growth factor of the NTF family. NGF is responsible for the survival and maintenance of specific neuronal subpopulation phenotypes in peripheral neurons and basal forebrain cholinergic during development and maturation (Rodríguez-Tébar et al., 1992, p. 35; Indo, 2018). NGF binds to and activates its high-affinity NTF family receptor TrkA on neurons and is internalized into responsive neurons. The NGF/TrkA complex is then transferred retroactively through the axon to the neuronal cytosol (Hirose et al., 2016). This movement of NGF from the axon tip to the soma is thought to be associated with long-distance signaling in neurons. It has been shown that NRG1 is also involved in fear extinction. NRG1 can exert its biological function by activating the ErbB4 protein, and the NRG1-ErbB4 signaling pathway plays an essential role in axon guidance, glial cell development, axon myelination, synapse formation, synaptic plasticity, and neural survival (Chen et al., 2022b). This pathway regulates PV neuronal network plasticity-mediated fear extinction in IL mPFC (Chen et al., 2022b). Specifically, fear extinction induces sustained plasticity in the PV neuronal network, as evidenced by elevated expression of NRG1 in IL but not PL regions.

In addition, neurotrophic factor 3 (NTF-3) is the third characterized member of the NTF family, except for NGF and BDNF. de Miranda et al. proposed that NTF-3 is a potential pharmacological target for emotional disorders and can regulate monoamine neurotransmitter transmission, BDNF signaling, synaptic plasticity, and neurogenesis (de Miranda et al., 2020). One hour after the last extinction training was considered as the consolidation phase of fear memory extinction when a local infusion of NTF-3 in the mPFC of mice promoted the LTP of IL and significantly enhanced the extinction process of fear memory. Furthermore, this enhancement effect could be blocked by peripheral administration of SL327, which inhibits the phosphorylation process of the extracellular signal-regulated kinase (ERK). This implies that NT-3/TrkC and ERK signaling pathways may be essential in fear extinction (D'Amico et al., 2017). In addition, NT-3/TrkC is also involved in the process of glutamate release (Aghajanian and Rasmussen, 1989). Thus, the facilitation of fear extinction by NT-3 may be influenced by the modulatory effects of glutamatergic neurotransmission.

#### 4.2.9. Acid-sensing ion channel 1a

Acid-sensing ion channel 1a (ASIC1a) is a class of extracellular proton-gated cation channels that allow selective passage of cations in the CNS, mainly in the HPC, AMY, ACC, somatosensory cortex, and striatum (Waldmann et al., 1997). ASIC1a is located in the postsynaptic membrane and can be activated by protons released from acidic vesicles during synaptic transmission to participate in synaptic signaling and plasticity, playing an essential role in learning and memory. Previous studies have shown that ASIC1a is associated with synaptic plasticity, including LTP and LTD. Knockdown of the ASIC1a gene impairs LTP in the AMY. Depending on the different expression levels of ASIC1a in vHPC, ASIC1a exhibits two opposite effects (Wemmie et al., 2004; Jiang et al., 2017). On the one hand, the deletion of ASIC1a leads to diminished conditioned fear memory. On the other hand, overexpression of ASIC1a promotes fear extinction (Wemmie et al., 2004). Specifically, ASIC1a in vHPC may mediate the fear extinction process through Ca^2+^ influx, membrane depolarization, enhanced postsynaptic NMDAR anti-synaptic properties, increased learning-dependent BDNF expression, and subsequent reverse projection of BDNF signals from vHPC to the IL (Wang et al., 2018). The role of ASIC1a indirectly reflects the importance of NMDA signaling.

#### 4.2.10. Epigenetic regulation

Epigenetic regulation refers to the process of gene expression changes that alter only the structure of chromatin but not the nucleotide sequence, including histone modifications (Cholewa-Waclaw et al., 2016), DNA methylation (Su and Tsai, 2012), RNA modifications (Widagdo et al., 2016), non-coding RNA-mediated regulation (Jung and Goldman, 2018), and other processes. Epigenetic regulatory processes are closely associated with learning and memory. Alterations in epigenetic mechanisms are also associated with many mental disorders, neurodevelopmental disorders, and neurodegenerative diseases (Cholewa-Waclaw et al., 2016). The consolidation of fear memory requires the involvement of protein synthesis. This process also involves the coordinated transcription of specific genes encoding learning and memory-related transcription factors, cytoskeletal proteins, and neurotransmitter receptors, among others (Alberini, 2009). The N-terminal tail of histones can be modified such as acetylation, methylation, and phosphorylation. Histone modifications play an essential role in gene regulation (Jenuwein and Allis, 2001). Studies have shown that epigenetic regulation (e.g., histone acetylation) can affect fear extinction. Histone acetylation is an essential epigenetic modification mechanism for cellular regulation of gene expression, and its abnormal regulation is associated with various inflammatory diseases (Brown et al., 2014). Studies have shown that enhancing gene transcription by increasing histone acetylation can improve fear extinction memory. Acetylation and deacetylation of lysine residues in histone tails are regulated by opposite effects from histone acetyltransferase (HAT) and histone deacetylase (HDAC). For example, genetic or pharmacological inhibition of HDAC by administration of HDAC inhibitors (e.g., vorinostat/octanoylanilide isohydroxamic acid and valproic acid) can increase histone acetylation and thus enhance fear extinction (Zovkic and Sweatt, 2013). As previously mentioned, BDNF is closely associated with synaptic transmission and synaptic plasticity, and BDNF transcription is also epigenetically regulated. Enhanced histone acetylation was observed in the promoter region of BDNF exon IV after successful fear extinction. Thus, BDNF is also considered an important downstream target for HDAC inhibitors to promote fear memory extinction (Bredy et al., 2007). The BET family of bromodomain co-activator proteins also plays a crucial role in regulating gene transcription as acetylated histone “readers”. It has been shown that the BET protein inhibitor JQ-1 selectively disrupts the extinction of the remote fear memory (14 days after the establishment of the fear conditioning). This may be because remote fear extinction training upregulates IGF-2 mRNA and protein levels in ACC but not HPC, and JQ-1 blocks IGF-2 expression in ACC that is increased by remote fear extinction training (Duan et al., 2020).

Other studies have identified DNA methylation (DNAm) as a critical epigenetic mechanism associated with fear extinction (Whittle and Singewald, 2014; Agís-Balboa et al., 2017). More than 20 different base modifications have been identified in DNA. Several studies have been conducted on 5-methylcytosine (5 mC), 5-hydroxymethylcytosine (5 hmC), and N^6^-methyl-2′-deoxyadenosine (m^6^dA). Li et al. suggested that learning-induced accumulation of m^6^dA in postmitotic neurons is associated with increased gene expression related to m^6^dA and further targeted the epigenetic mechanisms of m^6^dA during fear learning (Li et al., 2019). Infusing N6amt1 shRNA into the IL mPFC before extinction training found that fear extinction memory was severely impaired in mice, whereas infusion into the PL was not. N^6^amt1-mediated accumulation of m^6^dA in the IL PFC may play an essential role in regulating the formation of fear extinction memory (Li et al., 2019).

N^6^-methyladenosine (m^6^ A) and N^6^,2′-O-dimethyladenosine (m^6^ Am) are two mRNA modifications that regulate RNA metabolism, translation, selective shearing, and non-coding transcription processes. The former is considered eukaryotes' most abundant internal mRNA modifier base (Linder et al., 2015). Studies have validated the great potential of m^6^ A and m^6^ Am in measuring stress and stress-related psychiatric disorders. For example, overexpression of m^6^ Am in synaptic and neuronal regulation genes was observed in cortical regions of mice experiencing acute stress exposure. Furthermore, m^6^ Am also exhibits region-specific characteristics. Region specificity refers to brain regions performing different functions and showing different activation levels at different cognitive stages. Specifically, in two brain regions (PFC and AMY) highly involved in stress processes, RNA methylation exhibits a decrease in the PFC and an increase in the AMY (Engel et al., 2018). Recently, Chang et al. first suggested that changes in RNA methylation levels may underlie contextual fear memory generalization and differentiation, which may result from m^6^ A regulation in glutamatergic neurons in the HPC. Although no studies have been found that directly elucidate the role of RNA methylation in fear extinction comprehensively, the above evidence suggests that RNA methylation likely facilitates or enhances fear memory extinction by modulating changes in the molecular mechanisms underlying memory (Marshall and Bredy, 2019).

Abnormal fear is associated with a variety of fear and stress disorders, such as phobias, depression, social phobia, and PTSD. A typical characteristic of these disorders is the inability to appropriately suppress or subdue the fear response after the fearful stimulus has disappeared. Currently, treatments for stress-related disorders include psychotherapy (exposure therapy and cognitive-behavioral therapy), pharmacotherapy (beta-blockers, benzodiazepines, and selective 5-hydroxytryptamine reuptake inhibitors), neuromodulation techniques (transcranial magnetic stimulation and transcranial direct current stimulation), etc.

## 5. Mechanisms of fear relapse

In addition to the mechanisms of fear memory mentioned above, fear relapse is also an important issue that is worthy to discuss (Lee and Kaang, 2023). On the one hand, successful extinction of fear memory is not the elimination or renewal of the original fear memory, but the generation of new secure memory formation competing with the original memory. This determines that fear memory may recur in a variety of contexts. On the other hand, exposure therapy based on extinction training is currently one of the main clinical treatments for phobias and anxiety disorders, but it has a high relapse rate (Goode and Maren, 2014). Therefore, it is of clinical importance to elucidate the mechanisms of fear relapse. Fear relapse consists of several factors, such as exposure to a new context (renewal) and time passing (spontaneous recovery; Chen et al., 2017; Lacagnina et al., 2019; Lee and Kaang, 2023).

Considering that renewal refers to the expression of fearful emotions in contexts other than the fearful context, the HPC, which is closely related to contextual memory, is crucial for the renewal of fear memory. For example, inactivating the HPC using pharmacological techniques impairs the renewal of extinguished memory (Corcoran and Maren, 2001; Ji and Maren, 2008). The important role of the HPC in fear memory renewal has also been confirmed in human studies. It was found that individuals with stronger skin conductance responses in novel contexts exhibited enhanced effective connectivity between hippocampal activation areas and fear extinction-related brain networks (Hermann et al., 2016).

In addition to shedding light on the mechanisms underlying fear memory renewal from a broad range of brain regions, existing studies have also focused on the molecular mechanisms behind renewal. Dopamine is an important regulator of fear memory and is mainly located in the ventral tegmental area (VTA) and substantia nigra (SN; Salinas-Hernández and Duvarci, 2021). Dopamine neurons in the VTA project to the nucleus accumbens (NAC), which is responsible for supplying dopamine. During fear extinction, dopamine release is increased in the NAC, and the extinction of fear memory is impaired by the pharmacological blockade of dopamine release (Badrinarayan et al., 2012). D2 receptors in the NAC may play an important role (Wise, 2009). In recent years, dopaminergic neurons projecting from the SN to the dorsal striatum have also been implicated in emotion. It has been found that activation of dopaminergic neurons in SN during fear extinction not only enhances the extinction of fear memory but also prevents the renewal of fear memory (Bouchet et al., 2018). D1 receptors in the dorsal striatum may play an important role in the renewal of fear memory. For example, injecting D1 agonists into the dorsal striatum before fear extinction significantly inhibits fear memory renewal, as evidenced by less freezing in a new context (Bouchet et al., 2018). In short, clarifying the neurobiological mechanisms of renewal can help develop effective therapeutic targets and enhance the long-term efficacy of a range of treatments such as exposure therapy.

The re-emergence of fear responses after a period of time in individuals who have received extinction training is known as spontaneous recovery. Both spontaneous recovery and fear memory extinction processes may involve AMY, mPFC, and HPC. It was shown that mice treated with acute nicotine showed increased spontaneous recovery of extinguished fear memory, exhibiting a significant increase in the number of immunoreactive cells in HPC and BLA and a decrease in immunoreactive cells in PL (Kutlu et al., 2016). This suggests that spontaneous recovery may be closely related to increased activity in the HPC and BLA and suppression of activity in cortical areas. As its mechanisms, spontaneous recovery is closely related to synaptic plasticity. This is because spontaneous recovery implies that the acquired fear extinction memory was not successfully transformed into long-term memory (Eisenberg and Dudai, 2004; Lee and Kaang, 2023). Blocking endogenous histone deacetylases (Hdacs) promotes synaptic plasticity which is a mechanism necessary for the transition from short-term to long-term memory. It was found that the knockdown of Hdac2 in PV^+^ cells can reduce the spontaneous recovery of fear memories over time (Lavertu-Jolin et al., 2023). There are several intriguing insights into the mechanisms of spontaneous recovery from fear, yet there is still much that remains unclear.

## 6. Perspective on the treatment of trauma and fear-related disorders

### 6.1. Psychotherapy

Psychotherapy is one of the effective methods available to treat trauma and fear-related disorders such as phobias (Ma et al., 2021). Psychotherapy can be preferred for children and mild patients (Cohen et al., 2000). Exposure therapy is also an effective method of treating trauma and fear-related disorders (Rothbaum and Schwartz, 2002). This therapy is based on Foa and Kozak's Emotional Processing Theory (Rauch and Foa, 2006; Alpert et al., 2021). Current effective measures for exposure therapy include setting multiple exposure targets and combining different exposure targets in a single exposure experiment to focus on uniformly deepening fear extinction (Rescorla, 2006); eliminating the patient's safety behaviors early (Rachman et al., 2008); and encouraging the patient to express the emotion out loud and to name the emotion (Kircanski et al., 2012). The main psychological therapies for social phobia are cognitive-behavior therapy (CBT; Alpert et al., 2021). CBT advocates a combination of cognitive and behavioral correction, with cognitive correction mainly using the analysis of the causes and pathological substance of the distorted beliefs and factors that hinder normal psychological development and encouraging the patients to establish correct beliefs (Škodlar et al., 2012; Beck et al., 2015). At the same time, behavioral therapy principles are used to make the new cognitions put into practice, verify and consolidate, and accelerate the elimination of social terror. One of the well-established CBTs is cognitive-behavioral group therapy (McEvoy, 2007).

### 6.2. Pharmacotherapy

Effective drugs for the treatment of these disorders are tricyclic antidepressants (TCAs; Zohar and Westenberg, 2007; Puetz et al., 2015), reversible and irreversible monoamine oxidase inhibitors (MAOIs; Liebowitz et al., 1990; Chamberlain and Baldwin, 2021), benzodiazepines (Steenen et al., 2016), selective serotonin reuptake inhibitor (SSRIs; Zohar and Westenberg, 2007), and antiepileptic drugs (Berlin, 2007; Gasparyan et al., 2022). TCAs and MAOIs are effective for treatment but have high side effects, thus limiting their use. Two small randomized trials found that the highly potent benzodiazepines alprazolam and clonazepam were effective as monotherapy in reducing symptoms of social phobia (Knijnik et al., 2008). Clonazepam is a slower-acting, long-acting drug more commonly used for social phobia than other benzodiazepines. However, high-potency benzodiazepines are also contraindicated for long-term use due to the dependence and addiction associated with long-term use (Chouinard, 2004). SSRIs (e.g., paroxetine and sertraline) are effective in treatment but have few side effects, which are the only class of antidepressants approved for the treatment of PTSD (Akiki and Abdallah, 2019). Pregabalin (an antiepileptic drug) was approved in Europe in 2005 for treating anxiety disorders. Recently, cannabidiol (CBD) was also approved by the FDA for an Investigational New Drug Clinical Trial Application (IND; Lézard, 2021). In future, CBD may be known as an effective drug for treating PTSD and other stress-related disorders. In addition, 3,4-methylenedioxy-methamphetamine (MDMA) induces 5-hydroxytryptamine release by binding to the presynaptic 5-hydroxytryptamine transporter, thereby enhancing the elimination of fear memory (Rudnick and Wall, 1992). An analysis of data from six phase II clinical studies showed that MDMA was safe and efficacious in the treatment of PTSD, providing a rationale for MDMA in the treatment of PTSD. Mitchell et al. (2021) further conducted a phase III clinical study and found that MDMA-assisted treatment is potentially beneficial for patients with PTSD and may also be more effective than SSRIs (Mitchell et al., 2021). Sufficient replication trials to be conducted before clinical use can be introduced. In summary, more and more drugs are being developed for stress-related disorders. Considering the adverse effects of drugs, the combination of pharmacotherapy and psychotherapy seems to be an effective way to treat stress-related disorders (Leichsenring et al., 2022).

### 6.3. Neuromodulation techniques

Non-invasive brain stimulation technique modulates neural activity through synaptic plasticity and provides a potential treatment for many neuropsychiatric disorders. Previous studies have shown that non-invasive brain stimulation techniques such as transcranial magnetic stimulation (TMS) and transcranial direct current stimulation (tDCS) have clinical value for the treatment of stress-related disorders such as anxiety disorders and PTSD (Clark et al., 2015; Wout et al., 2017; Philip et al., 2019; Gouveia et al., 2020). For example, high-frequency (20 Hz) repetitive transcranial magnetic stimulation (rTMS) of the left and right dlPFC was effective in relieving the symptoms of PTSD, and stimulation of the right side was more effective than the left side (Boggio et al., 2010). A meta-analysis study has shown that both excitatory and inhibitory rTMS of the right dlPFC can reduce PTSD-like (Romero Lauro et al., 2014) symptoms, and the treatment effect is longer lasting with continuous stimulation for 2–4 weeks (Kan et al., 2020). Theta burst stimulation (TBS) is a new TMS stimulation protocol based on the spontaneous theta rhythm of the hippocampal memory system and consists of pulses of high-frequency stimulation (Cheng et al., 2021). Studies showed that iTBS significantly improved PTSD-like symptoms, enhanced social and occupational functioning, and reduced depressive tendencies (Philip et al., 2019). tDCS is also a non-invasive brain stimulation technique that modulates the resting membrane potential of neurons by applying a constant, low-intensity direct current microcurrent to specific brain regions, thereby modulating the body's cognition and mood (Nitsche et al., 2008). The anodic electrodes stimulate the cortical excitability of the region (Romero Lauro et al., 2014). Cortical excitability increases in areas stimulated by anodal electrodes and decreases in areas stimulated by cathodal electrodes (Fregni et al., 2006). Self-reported PTSD-like clinical symptoms and psychophysiological arousal in veterans with PTSD can be significantly improved by combining virtual reality (VR) and tDCS (Van'T Wout-Frank et al., 2019). There have been many studies supporting the effectiveness and safety of non-invasive brain stimulation for the treatment of anxiety disorders (Sagliano et al., 2019; Vicario et al., 2019, 2023; Sá et al., 2023). However, TMS techniques still have some limitations, requiring long-term continuous stimulation of the patient's brain nerves and high stimulation accuracy (Cheng et al., 2021). In addition, the current results are highly heterogeneous (Cheng et al., 2021), which makes it impossible to make accurate judgments about its efficacy and optimal stimulation parameters. In future, a larger sample size is needed to demonstrate the effectiveness and safety of non-invasive brain stimulation (Gouveia et al., 2020).

## 7. Summary and outlook

Fear has long received widespread attention as a fundamental emotional experience. Abnormal fear memories can cause a series of trauma and fear-related neuropsychiatric disorders, which suggests that an inability to extinguish fear is one possible mechanism of these disorders. This review has systematically summarized the categories and processes of fear memory. The fear memory models and their variants were discussed to help researchers better explore the mechanisms of fear memory properly. The review then thoroughly discussed the brain regions (mainly the triple network consisting of mPFC, AMY, and HPC) and inter-regional neural circuits involved in fear extinction, and explored the molecular mechanisms from multiple perspectives. Regarding treatments, psychotherapy (e.g., exposure therapy, cognitive-behavior therapy) and pharmacotherapy (e.g., SSRIs, antidepressant drugs) are used primarily to treat stress-related disorders. Traditional psychotropic medicines and drug delivery methods are widely used but poorly targeted and prone to side effects. Non-invasive brain stimulations, such as TMS and tDCS, have been applied in treating psychiatric disorders. Still, it has not been combined with the pathological basis of neural circuit abnormalities to achieve precise treatment of stimulation sites and parameters. Therefore, the neurobiological mechanism of fear memory extinction, especially the neural circuit and molecular mechanism of fear memory extinction, is a central “pain point” in cognition and brain disease research. How to specifically intervene in the abnormal neural circuits of fear extinction according to the characteristics of symptoms is essential for the precise treatment of related diseases. Recently, optogenetics and chemical genetics have shown a powerful role in parsing the neural mechanisms underlying specific cognitive behavioral patterns (e.g., memory processes) and abnormal neuropsychiatric disease phenotypes (e.g., trembling in response to fear). Numerous studies have successfully modulated the expression and extinction of fear memory in mice using optogenetic and chemical genetic approaches (Canto-de-Souza et al., 2021; Gu et al., 2022). In addition, acoustic and magnetic thermal genetics, as a minimally invasive neuromodulation technique with high spatial and temporal resolution, can achieve precise control of specific brain regions and has application prospects in the clinical treatment of trauma and fear-related diseases. Relative research has laid a solid foundation for achieving the regulation of brain regions associated with fear extinction (Munshi et al., 2017; Duque et al., 2022; Xian et al., 2023; Yang et al., 2023). It can be seen that the application of the above neuromodulation techniques for the treatment of stress-related disorders such as PTSD in humans is becoming possible.

In summary, a plausible explanation for fear extinction needs to be considered from different perspectives. Regarding neural mechanisms, existing studies have increasingly emphasized the critical role of the tripartite dynamic network among mPFC, AMY, and HPC in fear extinction. Recent studies have started to consider these brain regions as neural networks of fear extinction and to carry out an in-depth investigation at the level of neural circuits and their molecular mechanisms. However, the brain remains an enigma, and future studies can deeply explore the role of brain regions, neural circuits, specific neuronal clusters, and their molecular mechanisms to clarify the connections between different dimensions of the fear extinction process. Second, convincible experimental results must be based on a standard and rigorously designed paradigm. Therefore, future research will require continuous refinement of existing paradigms or models and new paradigms to match better the symptoms or problems of specific stress and post-traumatic disorders. Technological innovations and developments can provide new principles, ideas, and approaches to address complex issues in the field. In addition to applying existing neuromodulation techniques, such as gene editing and viral tracer techniques, a rational combination of existing or newly developed tools can be considered in future (Gu et al., 2022; Liu et al., 2023; Yu et al., 2023). This will not only provide an effective way to explore the underlying mechanisms of the fear extinction process but also bring more possibilities to elucidate the functional connectivity of the brain and even contribute to the clinical treatment of various neurological diseases. In conclusion, the neurobiological mechanisms of fear extinction are expected to be resolved in more detail, which is necessary for developing public mental health.

## Author contributions

YL and WZ wrote the article and outlined the manuscript. BQ, LW, and XH provided detailed guidance throughout the article. All authors read and approved the final manuscript.
